# Molecular markers reveal diversity in composition of *Megastigmus* (Hymenoptera: Megastigmidae) from eucalypt galls

**DOI:** 10.1002/ece3.6791

**Published:** 2020-09-25

**Authors:** Ngoc Hoan Le, Helen F. Nahrung, Jess A. T. Morgan, Steven Ogbourne, Simon A. Lawson

**Affiliations:** ^1^ Forest Industries Research Centre University of the Sunshine Coast Sippy Downs QLD Australia; ^2^ Department of Agriculture and Fisheries EcoSciences Precinct Brisbane QLD Australia; ^3^ GeneCology Research Centre School of Science and Engineering University of the Sunshine Coast Sippy Downs QLD Australia

**Keywords:** gall associates, *Leptocybe*, *Megastigmus lawsoni*, *Megastigmus zebrinus*, parasitoids

## Abstract

Since outbreaks of the invasive blue gum chalcids *Leptocybe* spp. began, the genus *Megastigmus* (Hymenoptera: Megastigmidae) has been increasingly studied as containing potential biocontrol agents against these pests. *Megastigmus* species have been collected and described from Australia, the presumed origin of *Leptocybe* spp., with *M. zvimendeli* and *M. lawsoni* reported as *Leptocybe* spp. parasitoids established outside of Australia. Parasitic *Megastigmus* have been reported to occur locally in the Neotropics, Afrotropic, Palearctic, and Indomalaya biogeographic realms, and in many cases described as new to science. However, molecular tools have not been used in studying parasitic *Megastigmus*, and difficulties in morphological taxonomy have compromised further understanding of eucalypt‐associated *Megastigmus* as well as the *Megastigmus*‐*Leptocybe* association. In this study, we used molecular markers to study the species composition and phylogeny of *Megastigmus* collected from eucalypt galls in Australia and from *Leptocybe* spp. galls from South Africa, Kenya, Israel, China, and Vietnam. We record thirteen discrete species and a species complex associated with eucalypt galls. A summary of morphological characters is provided to assist morphological delimitation of the studied group. A phylogeny based on 28S rDNA identified species groups of importance to *Leptocybe* spp. biocontrol agents from four clades with nine species. Relationships between *Megastigmus* from eucalypt galls and their phytophagous congeners were unresolved. Further molecular work is needed to clarify the identity of many species.

## Graphical abstract

This study used molecular markers to investigate the species composition and phylogeny of *Megastigmus*, the increasingly important biocontrol agents against the worldwide invasive blue gum chalcids *Leptocybe* spp. We recorded thirteen discrete species and a species complex associated with eucalypt galls and provided a summary of morphological characters to assist morphological delimitation of the studied group. A phylogeny based on 28S rDNA was provided, and relationships between *Megastigmus* from eucalypt galls and their phytophagous congeners were discussed

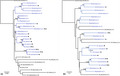

## 1 INTRODUCTION

*Megastigmus*, comprising 145 described species (Noyes, 2020), is the largest genus in the newly recognized family Megastigmidae (Janšta et al., 2018). Members of the genus are most abundant in the Palearctic and Australia (Grissell, 1999), with the latter the most likely origin of the family (Janšta et al., 2018). *Megastigmus* encompasses diverse feeding habits, from strict phytophagy to strict entomophagy, with facultative entomophagy, also referred to as partial phytophagy or inquilinity, an “intermediate form” between the two (Grissell, 1999). Before 2000, literature of non‐Australian *Megastigmus* predominantly described phytophagous species, while records from Australia largely comprised of entomophagous *Megastigmus* (Auger‐Rozenberg et al., 2006; Grissell, 1999; Roques & Skrzypczyńska, 2003). However, entomophagy was mostly inferred from observations of emergence from galls, without clear data discriminating parasitic, inquiline, and hyper‐parasitic behavior (Bouček, 1988; Grissell, 1999).

*Leptocybe* (Hymenoptera: Eulophidae) is a genus of galling insects of Australian origin, with two invasive species causing damages to the eucalypt forestry worldwide under the cryptic name *L. invasa* (Dittrich‐Schröder et al., 2018; Kim, 2008; Nugnes et al., 2015). *Megastigmus* spp. associated with eucalypt galls have gained increasing interest since *L. invasa* was first described (Le, Nahrung, Griffiths, & Lawson, 2018; Mendel, Protasov, Fisher, & La Salle, 2004; Viggiani, Laudonia, & Bernardo, 2001). Six new *Megastigmus* species were described from Australia (Doğanlar, 2015; Doğanlar & Hassan, 2010), of which *M. zvimendeli* and *M. lawsoni* were released and established as biocontrol agents of *L. invasa* in Israel (Mendel et al., 2017). *Megastigmus zebrinus* was described from specimens from South Africa and Australia (Grissell, 2006) and was later reported to occur in Thailand and Argentina (Doğanlar, 2015). Several species of *Megastigmus* have been recorded as associated with *Leptocybe* spp. in the Indomalaya, Palearctic, Afrotropic, and Neotropic biogeographic realms, and in many cases described as new species to science (Le et al., 2018).

Discrimination of *Megastigmus* associated with *Leptocybe* spp. has so far relied largely on morphology (Doğanlar, 2015; Doğanlar & Hassan, 2010). This has caused uncertainty and impeded further understanding of phylogenetic relatedness, since variation in color and sizes of specimens has long been known to challenge taxonomists, particularly those working with Australian species (Bouček, 1988; Milliron, 1949). While molecular markers have been used in systematic studies and species delimitation of the Palearctic and Afrotropical fauna (Auger‐Rozenberg et al., 2006; Roques, Copeland, Soldati, Denux, & Auger‐Rozenberg, 2016), continued use of these markers is expected to assist species delimitation and give further insight into the phylogeny of this genus. Incorporation of generated DNA sequences with published molecular data is expected to provide knowledge of the relationships between phytophagous and entomophagous species, and between Australian and non‐Australian *Megastigmus* (Le et al., 2018; Roques et al., 2016).

Here we present a study of eucalypt‐associated *Megastigmus*, with a focus on species of potential biocontrol use against invasive *Leptocybe* spp. Research materials included *Megastigmus* specimens from eucalypt galls collected in Queensland (QLD), New South Wales (NSW), Australian Capital Territory (ACT), Victoria (VIC), and, where possible, specimens associated with *Leptocybe* spp. galls in their exotic ranges provided by international colleagues. Species delimitation and phylogenetic reconstruction were completed using a combination of the Clyde‐Bonnie fragment of mitochondrial DNA coding cytochrome c oxidase subunit 1 (COI mtDNA) and the partial nuclear DNA fragment coding 28S ribosomal RNA (28S rDNA). This study is the first to report a molecular sequence comparison of gall‐associated *Megastigmus* in the eucalypt gall system.

## 2 MATERIALS AND METHODS

### 2.1 Insect collection

Galled eucalypt material was collected in road‐side surveys in QLD, NSW, ACT, and VIC between March 2014 and June 2019. Parts of eucalypt plants bearing galls on young shoots or leaves were collected and transferred to the laboratory within seven days of collection. Galls were placed in zip‐lock bags and stored in a cooled insulated box or fridge (4°C) during transportation and then transferred to separate plastic vials (Φ30 mm × H100 mm) containing moistened tissue paper. These vials were kept in a controlled temperature cabinet, at 25 ± 2°C, 50% to 70% RH, and emerged insects were collected every 2–3 days over ~30 days. Emerged *Megastigmus* were transferred to glass vials (Φ11.6 mm × H32 mm) containing 100% ethanol (volume of insect: ethanol <1:10) and stored at −20°C. Gall types bearing *Megastigmus* emergence were recorded and preliminarily sorted by gall morphology (see Appendix S1). Individual wasps were examined, photographed, and DNA was extracted when (a) specimens emerged from material from a new collection site; (b) specimens emerged from the same collection site but from different gall types or different eucalypt species; or (c) specimens appeared superficially different from those collected in (a) and (b). Species of *Bootanomyia* spp., characterized by the knobbed stigmal vein and exerted ovipositor like *Megastigmus* spp. but with a metallic body color (Bouček, 1988; Doğanlar, 2011), were also collected and included in DNA analyses but were not examined further morphologically.

### 2.2 DNA extraction, PCR, and sequencing

DNA was extracted from entire insect bodies (for small specimens) or from the abdomen (for large specimens) using an ISOLATE II Genomic DNA Kit (Bioline, Eveleigh NSW, AUS), or a prepGEM^®^ Insect kit (ZyGEM, Hamilton, Aotearoa, NZ). DNA was eluted into either 20 or 40 µl extraction volume, depending on the size of specimens. Undiluted genomic DNA was used in PCR amplification reaction using MyTaq™ HS Red DNA Polymerase (Bioline, Eveleigh NSW, AUS). Total reaction volumes were 10 µl including DNA template (1 µl), primer (1 µl each, at 10 µM concentration), premixed 5 × buffer (2 µl), HSTaq DNA polymerase (0.1 µl), and H_2_O (4.9 µl). PCR thermo‐cycling was carried out in a Bio‐Rad T100 (Greenslopes, QLD, AUS) using the setup 95°C for 1 min then 35 cycles of 95°C for 1 min + 55°C for 1 min and 72°C for 1 min then 72°C for 5 min for final extension before holding at 10°C. When the primer 1775‐COI‐F was used, the annealing temperature was reduced to 50°C.

The primers used (Table 1) were either from previous work on *Megastigmus* (Roques et al., 2016; Scheffer & Grissell, 2003) or designed in the course of this study. The targets sequenced were a partial region of the COI mitochondrial DNA (the “Clyde‐Bonnie”) and a fragment from the D1 to D3 region of the nuclear 28S rDNA. The primer pairs 1775‐COI‐F/2773‐COI‐R (amplicon size 1,040 bp) and 28S‐D1F/28S‐D3R (amplicon size ca. 1,090 bp) (Boivin et al., 2014; Roques et al., 2016) were attempted first. The alternative combination 28S‐D1F/28S‐1059R (amplicon size 1,078 bp) was used to amplify the 28S fragment if initial amplification failed. For *M. zvimendeli* specifically, to avoid pseudogene co‐amplification, the target COI fragments were amplified by replacing 1775‐COI‐F with the upstream forward primer LCO1490 (Folmer, Black, Hoeh, Lutz, & Vrijenhoek, 1994). With this primer pair, the amplicon size (1,304 bp) was too long for completely overlapping forward and reverse read in Sanger sequencing, but the reverse primer gave clean capillary separation for the targeted fragment. Alternatively, the 1,304 bp COI fragments were obtained by manually assembling shorter fragments amplified using combinations of LCO1490 and 2773‐COI‐R with internal primers (Table 1).

**Table 1:** Names, sequences, and reference sources of primers used for DNA extractions

Primer name	Sequence	Reference
1775‐COI‐*F* (Forward) (Clyde)	CGAATAAATAATATAAGATTTTG	Scheffer and Grissell (2003)
LCO1490 (Forward)	GGTCAACAAATCATAAAGATATTGG	Folmer et al. (1994)
2222‐COI‐*F* (Forward)	ATATTTTAATTTTACCAGGATTTGG	Scheffer and Grissell (2003)
2399‐COI‐R (Reverse)	TGTAGCTGAAGTAAAATAAGC	Authors
2413‐COI‐R (Reverse)	TCATCTAAAAACTTTAATTCCTGT	Scheffer and Grissell (2003)
2773‐COI‐R (Reverse) (Bonnie)	GGATAATCTCTATATCGACGAGGTAT	Scheffer and Grissell (2003)
28S‐D1F (Forward)	ACCCGCTGAATTTAAGCATAT	Auger‐Rozenberg et al. (2006)
28S‐D3R (Reverse)	TAGTTCACCATCTTTCGGGTC	Auger‐Rozenberg et al. (2006)
28S−1059R (Reverse)	TTTCGGGTCCCAACGTGTAC	Authors

PCR products were visualized by electrophoresis on 1 × TBE and agarose gel with GelRed® (Biotium, California, USA). Products with a single band at the desired fragment size were sent to Macrogen Inc. (Seoul, ROK) for purification and Sanger sequencing. Alternatively, purification and sequencing reactions were conducted on‐site using ExoSAP‐IT (Thermo Fisher Scientific, MA, USA) and a BigDye Terminator v3.1 Cycle Sequencing Kit (Thermo Fisher Scientific). On‐site sequenced products were forwarded to the Australian Genome Research Facility (QLD, Australia) for electrophoresis capillary separation.

### 2.3 Sequence alignment and species delimitation

Sequence alignment was performed using the built‐in Geneious Alignment program in Geneious Prime (Biomatters, Auckland, NZ). The paired forward and reverse reads were aligned and edited to unambiguous sequences unless otherwise specified. Primer sequences were removed from reads, and multiple sequences were aligned using Geneious alignment algorithm (Global alignment with free end gaps, Cost Matrix 70% similarity, Gap open Penalty 12, Gap extension Penalty 6), validated by eye, and trimmed to equal lengths for subsequent analysis. Mitochondrial DNA was verified by checking for stop codons, which suggest the presence of pseudogenes. Stop codons were detected by applying the invertebrate mitochondrial genetic code and translating DNA sequences into amino acids. Sequences from two specimens identified to *M. manonae* contained single base positions with double peaks nested within regions of clear, unambiguous signal, which likely represent within‐individual mitochondrial copy differences and were therefore labeled with degenerative bases following the IUPAC ambiguity code.

Genetic species delimitation was determined using the mtDNA COI sequences using the web version of the Automatic Barcode Gap Discovery (ABGD) tool (Puillandre, Lambert, Brouillet, & Achaz, 2012) and the General Mixed Yule Coalescent (GMYC) method (Pons et al., 2006). ABGD examines the frequency distribution of pairwise differences to find the gap separating intragroup and intergroup differences and partitions the dataset by recursive application of a range of user‐given thresholds P (the maximum divergence of intraspecific sequences). The model of evolution was KP80, which is a common parameter in mtDNA‐based species delimitation (Boykin, Armstrong, Kubatko, & De Barro, 2012; Evans & Paulay, 2012). GMYC is a likelihood method that analyses the branching time against the difference in branching rates at the level of species and population. An ultrametric tree was generated using the Bayesian Evolutionary Analysis by Sampling Trees (BEAST) software package family (Bouckaert et al., 2019) version 2.6.3. Alignment of COI sequences obtained in the study was imported using the componential program BEAUTi, the selected substitution model was HKY + G+I (the first model determined using jModeltest for the analyzed dataset that is available for analysis in the software package), selected tree prior was Speciation: Yule Process (Gernhard, 2008), the maximum clade credibility (MCC) tree was generated using the program TreeAnnotator. GMYC analysis was performed using the function *gmyc* in the package splits (Ezard, Fujisawa, & Barraclough, 2017).

The identification of *Leptocybe* specimens to lineage B was based on the barcoding region and compared with published data (Dittrich‐Schröder et al., 2018). Specimens of *Leptocybe* lineage B obtained in this study grouped with *Leptocybe* sp. lineage B with >99.3% identity. Other specimens referred to as “local” *Leptocybe* sp. were identified by morphology (Kim, 2008; Mendel et al., 2004), including one specimen extracted for DNA, compared with available *Leptocybe* barcoding sequences and confirmed to be an unpublished *Leptocybe* species.

### 2.4 Phylogenetic inference

Placement of the sequences obtained in this study into a larger phylogeny was completed by incorporating sequences from Auger‐Rozenberg et al. (2006) and Roques et al. (2016). Sequences were selected to represent *Megastigmus* groups associated with different plant families (phytophagous species on Pinaceae, Cupressaceae, Rosaceae, Malvaceae, Rhamnaceae, Anacardiaceae). The outgroup was selected from the Palearctic *Bootanomyia*, which formed the closest sister group to the studied Australian *Megastigmus* and *Bootanomyia* (Janšta et al., 2018). A genome search using the MegaBLAST algorithm (Morgulis et al., 2008) returned sequences from the genome assembly accession GCA_900490025, species *B. dorsalis* (Bunnefeld, Hearn, Stone, & Lohse, 2018) with matching fragments to use as outgroup (see details in Appendix S2). The average pairwise distance between this outgroup with the eucalypt‐associated *Bootanomyia*, eucalypt‐associated *Megastigmus,* and phytophagous *Megastigmus* taxa were, respectively, 0.095, 0.098, and 0.112 for COI and 0.049, 0.044, and 0.049 for 28S DNA. Duplicate sequences were removed, including an entry of *Megastigmus zebrinus* (KU984706, Roques et al., 2016), which was identical to sequences of *M. zebrinus* obtained in this study. One taxon was randomly selected if more than one sequence existed for a species‐level taxon, similar to Roques et al. (2016). Alignments were trimmed to the same length for analysis.

Phylogenetic trees were inferred using the maximum likelihood method (ML) (Guindon et al., 2010) and Bayesian analysis (BA) (Huelsenbeck & Ronquist, 2001; Ronquist & Huelsenbeck, 2003). Data were partitioned into three blocks for COI and four blocks for the concatenated alignment (three codon positions and one for 28S DNA). ML analysis was performed on the Geneious Prime platform using the plugin PHYML 2.2.4 for 28S and RAxML 8.2.11 (Stamatakis, 2014) for partitioned datasets. ML branch support was calculated by bootstrap resampling 1,000 times. BA analysis was based on the program MrBayes 3.2.7 (Ronquist & Huelsenbeck, 2003). Analyses were terminated when the average standard deviation of split frequencies dropped below 0.01. The best fit model of evolution for each dataset was determined separately using the program Jmodeltest2 (Darriba, Taboada, Doallo, & Posada, 2012) for 28S and PartitionFinder 2.1.1 (Lanfear, Frandsen, Wright, Senfeld, & Calcott, 2016) for partitioned datasets.

The transition/transversion ratios were calculated and plotted using functions in the R‐based package SPIDER (Brown et al., 2012).

### 2.5 Morphological identification

Species identified using molecular evidence were labeled with numbers, including the four collected *Bootanomyia* spp. A minimal character set is provided for the collected *Megastigmus* species to assist future diagnostic works. Terminology follows Graham (1969), Bouček (1988), Gibson, Read, and Fairchild (1998), Roques and Skrzypczyńska (2003), and Doğanlar and Hassan (2010). Abbreviations of body characters follow Le, Nahrung, Lawson, and Morgan (2020). Microscopic observations and photographs were taken under a binocular microscope (NIKON SMZ800N) with attached digital camera (TUCSEN H500), resolution 2,584 × 1,936 pixels. Where relevant, sizes were measured in pixels by the software ImageJ 1.52a (National Institute of Health, USA) and calibrated using a stage micrometer (Carl Zeiss 5 + 100/100 mm). If curved, antennal flagellum and the exerted part of ovipositor sheath were measured along the curve, similar to Ôtake (1987).

Ethanol‐preserved insect specimens and DNA vouchers were deposited at the Insect collection, Queensland Department of Agriculture and Fisheries, Dutton Park, QLD, Australia.

## 3 RESULTS

### 3.1 DNA sequences

A total of ninety‐six specimens were extracted for DNA. Regarding COI, 81 specimens successfully provided COI sequences of the same length, 849 bp in the final alignment. The alignment included 38 unique COI sequences and was without alignment gaps. Adenine and thymine accounted for 74.2% of the total bases on average and 94.9% of third codon positions, in line with previous observations of the AT‐rich content of hymenopteran mtDNA (Clary & Wolstenholme, 1985; Crozier & Crozier, 1993). The number of variable sites was 234/849 (191 sites at the third codon position). The observed pairwise transition/transversion ratio (R) was 0.50, and the maximum pairwise distance was 11.78%. The best fit evolution model was TIM2 + G + I (*Transition Model 2* with gamma‐distributed among‐site rate variation and a proportion of invariable sites (Posada, 2003), BIC score 10,196, estimated Gamma shape parameter 0.165, proportion of invariable sites 0.468).

For the nuclear 28S rDNA, 93 specimens were successfully sequenced, providing 21 unique sequences of 856–857 bp in length, with the final alignment of 858 bp including gaps. The uncorrected pairwise distance ranged from 0.0012 to 0.0461, the observed transition/transversion ratio (R) was 1.65, and the average G + C content was 57.3%. The number of variable sites was 95/858. The best fit model of DNA evolution was TPM1 + G+I (3‐*parameter model,* with gamma‐distributed among‐site rate variation and a proportion of invariable sites (Kimura, 1981), BIC score 4,571, estimated gamma shape parameter 0.697, proportion of invariant sites 0.704).

**Figure 1:**
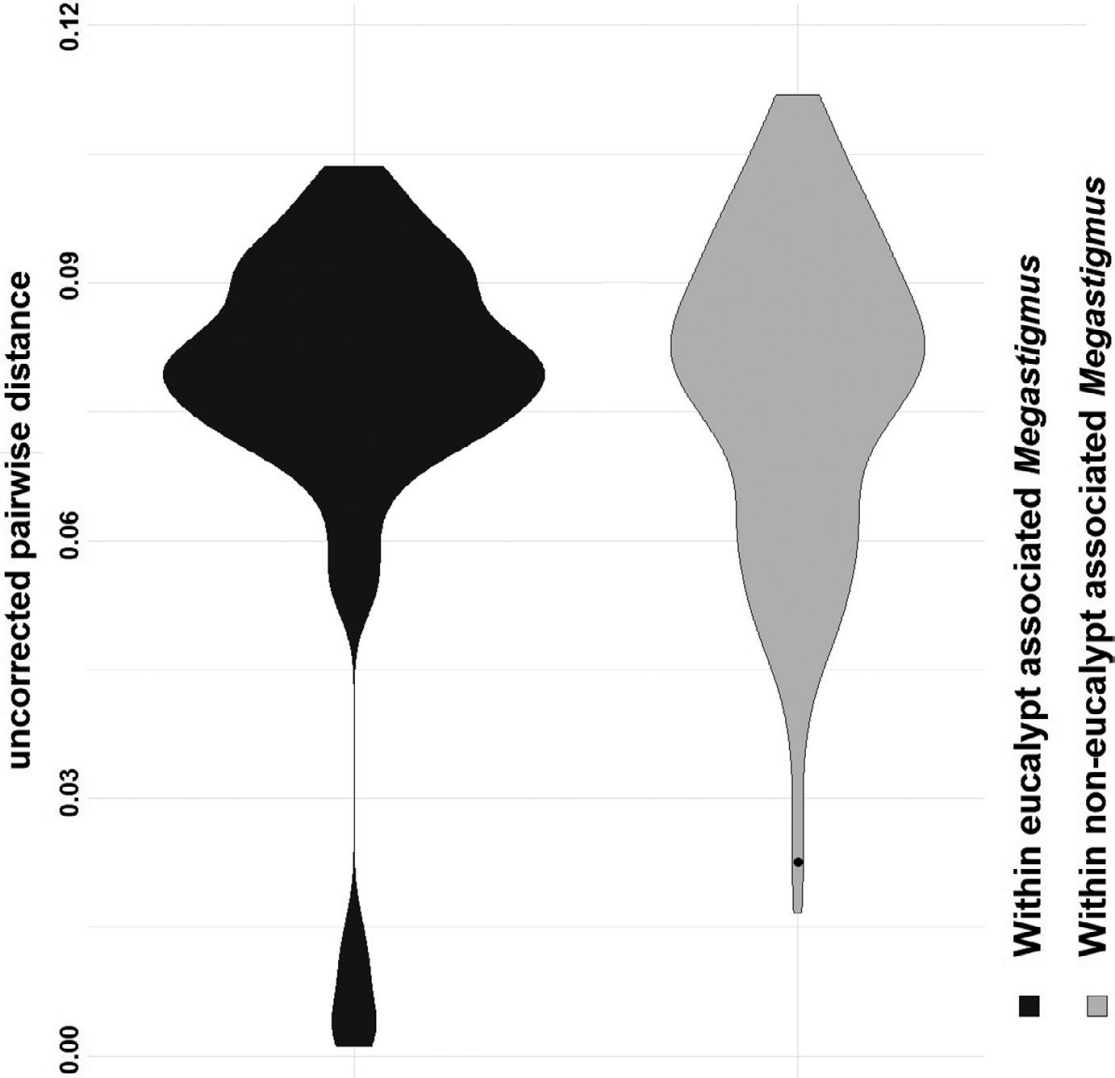
Violin boxplot illustrating the distribution of uncorrected pairwise distances between COI sequences of species associated with eucalypt galls (presumptive entomophagous) and phytophagous *Megastigmus*. Plot widths approximate the number of data points (number of pairwise comparisons). Distances values for the phytophagous group were interspecies except one data point (black dot, p‐distance 1.8% between two *M. aculeatus* specimens). Within‐group distances of species associated with eucalypt galls illustrated a clear barcoding gap separating intraspecies (lower) and interspecies (upper) values. All sequences are trimmed to 849 bp equal length. Sequences of phytophagous species were from Boivin et al. (2014) (see Appendix S2)

Uncorrected pairwise COI distances of eucalypt‐associated *Megastigmus* species (as presumptive entomophagous *Megastigmus*) and phytophagous *Megastigmus* are compared in Figure 1.

### 3.2 COI DNA based species delimitation

**Figure 2:**
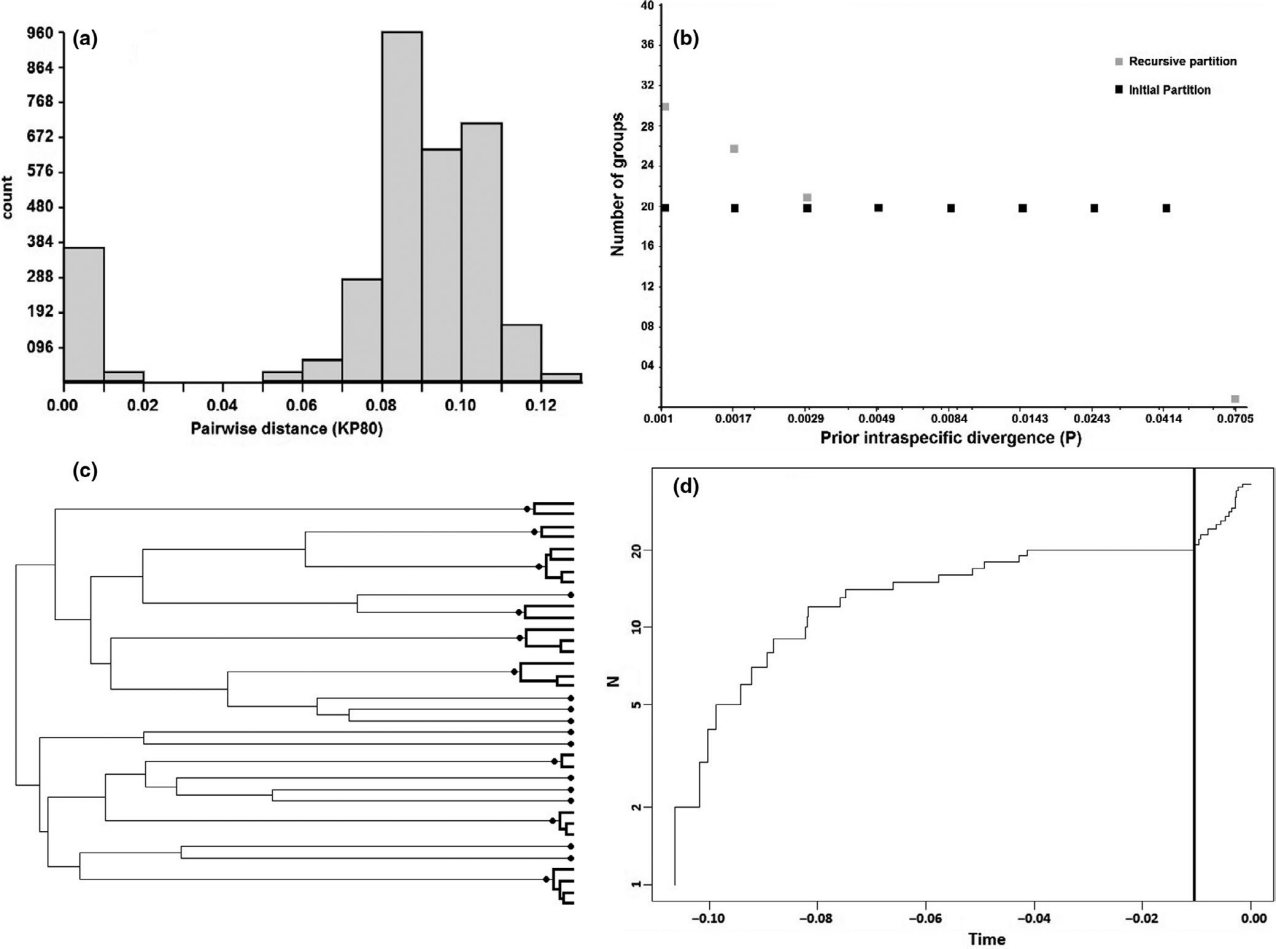
ABGD histogram (a), ABGD assignment of sequences into species group (b), GMYC assignment of sequences into 20 species based on a time‐measured phylogeny based on Bayesian analysis (c), and GMYC lineage‐through‐time plot (d). ABGD analysis based on 81 aligned sequences of 849 bp, *P*_min_ = 0.001, *P*_max_ = 0.12, Nbin = 13, model of evolution = KP80. GMYC tree was built using the program BEAST 2.6.3 using 35 unique COI sequences, Site model = Gamma, priors = Yule model and default settings

The ABGD analysis of the COI mtDNA alignment identified a clear barcoding gap between KP80 distance 0.02 and 0.05, that is, two sequences with KP80 distances of greater than 0.05 can be confidently assigned to different groups. The stable number of retrieved groups was 20, obtained when data were partitioned using P values from 0.0049 to 0.0414 (Figure 2). GMYC analysis suggested similar results with 20 Ml entities and the assignment of sequences to species identical to those suggested by ABGD (Figure 2). Based on the ABGD and GMYC analyses, the DNA sequences were assigned to 20 different operational taxonomic units comprising four *Bootanomyia* and sixteen *Megastigmus* species.

Aligning COI species delimitation with the morphological and distributional data, common *Megastigmus* species associated with *Leptocybe* spp. were identified. *Megastigmus pretorianensis* and *M. zebrinus* collected by colleagues in South Africa were identified based on collection locality and morphology (Doğanlar, 2015; Grissell, 2006). The *M. pretorianensis* specimens from Australia were identical to the South African specimens in 28S rDNA sequences and morphology. Its COI sequence (*M. pretorianensis* haplotype 2) was firmly assigned to the ABGD group for *M. pretorianensis*. *Megastigmus zvimendeli* and *M. manonae* were frequently collected from *Leptocybe* spp. galls in Queensland and northern NSW and were identified based on differential morphological diagnoses (Le et al., 2020). Specimens with females bearing one pair of scutellar setae could be assigned to the species complex *M. lawsoni* (Doğanlar & Hassan, 2010). *Megastigmus lawsoni 1*, *2,* and *3* were recognized by ABGD, and a possible fourth species, although not presented in ABGD and GMYC analysis, was regarded as *M. lawsoni 4*. This fourth species shared a unique 28S DNA which is different from the first three and an identical COI pseudogene differing from its functional gene by a deletion mutation. *M. lawsoni* specimens have one pair of scutellar setae (Doğanlar & Hassan, 2010), and males of all species in the complex bear a distinct black patch on the mesonotum around the transscutal articulation, but the rules of one pair of scutellar setae were seldom violated even within a population. Body ratio variation precluded further species identification; therefore, *M. lawsoni* was treated as a species complex.

Pairwise distances of COI and 28S datasets are provided in Appendix S3.

### 3.3 Morphological species delimitation

A set of morphological characters was proposed (Table 2) to assist in distinguishing female specimens for species identified by ABGD. *Megastigmus* sp. 5 and *Megastigmus* sp. 11 differed from other species by the distinct length of the ovipositor. Many species associated with *Leptocybe* spp. have the clava enlarged and width of the funicle segments strongly increasing from f1 to f7. These species can be further separated by the number of scutellar setae, as proposed by Doğanlar and Hassan (2010). Others have more filiform flagellum with a minor increase of funicle breadth apically, which is a characteristic closer to many phytophagous *Megastigmus* (Roques & Skrzypczyńska, 2003). These species can be further divided by the number of sensilla rows on funicle segments (Appendix S4).

**Table 2:** Morphological characters delimiting eucalypt gall‐associated *Megastigmus* in Australia (females, unless otherwise stated)

Species	Body color^1^	Antennal form	Scutellar setae^2^	Body size^3^	Antennal sensilla row	Ovipositor length^4^	*Leptocybe* spp. associate	Distinctive character
*M. lawsoni* ^5^ (complex)	1	Clavate	1	Small	1	Intermediate	Yes	1 pair scutellar setae, male with distinct black patch on mesonotum
*M. manonae* ^5^	2	Clavate	2	Small	1	Intermediate	Yes	2 pair scutellar setae, body darkly pigmented, *fu* **3** *.l* ≥ *fu* **2** *.l,* eye larger than *M. pretorianensis* (*eye.h*/*hea.hl* 0.72–0.85)
*M. pretorianensis* ^5^	2	Clavate	2	Small	1	Intermediate	Yes	2 pairs scutellar setae, body darkly pigmented, *fu* **3** *.l* ≤ *fu* ***2*** *.l,* eye smaller than *M. manonae* (*eye.h*/*hea.hl* 0.68 – 0.78)
*M. zvimendeli* ^5^	1	Clavate	2	Small	1	Intermediate	Yes	2 pair scutellar setae, body yellow/orange, *eye.h*/*hea.hl* (0.61–0.76) < *M. manonae*
*M. zebrinus* ^6^	1	Clavate	3a	Small—intermediate	1	Intermediate	Yes	3 pairs setae, *pol.l*/*ool.l* ≥ 3.5, interocullar bristles exist (Grissell, 2006)
*M*. sp. 1^5^	1, 3	Clavate	3a	Small—intermediate	1	Short	Yes	Ovipositor short ≤ 1.3x *gst.ll*
*M*. sp. 2	3	Filiform	3a	Large	2	Long	No	Ovipositor long, 2 rows sensilla
*M*. sp. 4	3	Intermediate	3a	Intermediate	2	Intermediate	No	2 rows of sensilla
*M*. sp. 5	4	Filiform	3a	Large	2	Long	No	Body black, long ovipositor
*M*. sp. 6	1	Clavate	3b	Small—intermediate	1	Intermediate	Yes	*pol.l*/*ool.l* ≤ 2, scutellar setae positioning 3b
*M*. sp. 8	3	Intermediate	3b	Intermediate	1	Intermediate	No	Scutellar setae positioning 3b
*M*. sp. 9	3–4	Filiform	3a	Intermediate	1	Intermediate	No	Antenna filiform, intermediate body size, scutellum and mesoscutum conspicuous black
*M*. sp. 10	1	Filiform	3a	Intermediate	‐	Intermediate	No	Stigma knob pointed
*M*. sp. 11	3	Filiform	3a	Intermediate—large	2	Average	No	2 rows sensilla, antenna long (1× *mss.ll*)

Note

^1^ Body color code 1: dominantly yellow, entirely yellow if examined with bare eyes; 2: Yellow with dark pigments scattered throughout body, most conspicuous when examining thorax area; 3. Yellow with conspicuous black areas, at least at propodeum and transscutal area. Antenna dark and hence antennal sensilla conspicuous; 4. Almost entirely black, nonglossy, most conspicuous at thorax.

^2^ Number and positioning of scutellar setae: 1, 2, 3: 3a = the distance between middle pair and anterior pair (*d1*) approximate that to the posterior pair (*d2*), 3b = *d1* ~ 2 × *d2*.

^3^ Classified using the combined gaster length + mesosoma length. Small: *gst.ll* + *mss.ll* ≤ 1.2 mm; intermediate: *gst.ll* + *mss.ll* 1.2 – 2 mm; large: *gst.ll* + *mss.ll* ≥ 2 mm

^4^ Classified using the ratio ovipositor length: gaster length. Short: *ovi.l*/g*st.ll* ≤ 1.3; long: *ovi.l*/*gst.ll* ≥ 2

^5^ Discussed in detail in Le et al. (2020)

^6^ Examination of *M. zebrinus* was based on ANIC and QM paratypes and South African alcohol preserved specimens

Extreme care should be taken for morphological identification of species that were observed to coemerge from the same galling material: *M. lawsoni* with *M. zvimendeli*; *Megastigmus* sp. 1 with *M. zvimendeli*; *M. manonae* with *M. zvimendeli* (in galls of *Leptocybe* sp. lineage B); *Megastigmus* sp. 6 with *M. pretorianensis* (in galls of a local, unreported *Leptocybe* sp.); *Megastigmus* sp. 1 with *M. manonae* (in multiple blister leaf galls). *Megastigmus* sp. 1 was found with two forms differing strongly in color and body sizes, in which the smaller form frequently associated with *Leptocybe* sp. lineage B. The association of conspecific males and females in this study was based on sequencing both males and females emerging from the same material, and in many cases, on sequencing the laboratory‐generated male offspring and linked with its female parent (Appendix S2). However, data on morphology of males were not presented for species delimitation as variation in size and form of males has not been fully investigated. The morphological characters presented in Table 2 were presented for females only, except for the distinct black patch in males of *M. lawsoni* (Le et al., 2020).

### 3.4 Phylogeny of eucalypt gall‐associated Megastigmus

**Figure 3:**
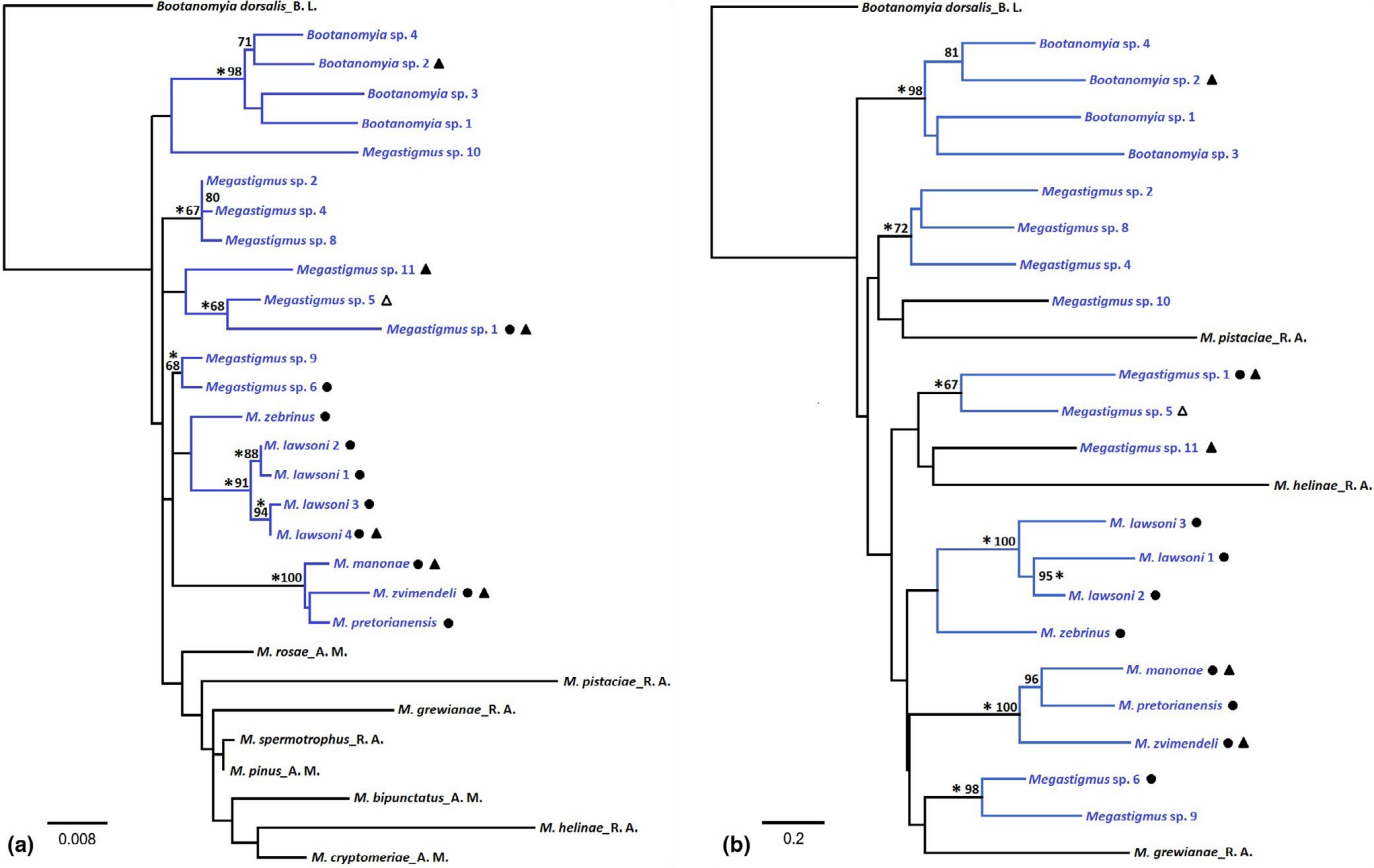
Phylogeny of *Megastigmus* and *Bootanomyia* species associated with eucalypt galls in reference to noneucalypt phytophagous *Megastigmus*. (a) 28S, 865 bp including alignment gaps, model of evolution TPM1 + G+I, using PHYML; (b) concatenated 28S and COI, 1,620 bp including gaps, model of evolution GTR + I + G, using RAxML, data partitioned into four blocks (28S + 3 codon positions). Analyses were performed in the Geneious Primer platform, and branch support was determined using 1,000 bootstraps. Taxa associated with *Leptocybe* spp. in Australia or *Leptocybe* spp. in their invasive range are marked with black dots. Taxa in blue were eucalypt gall associates. Nodes marked with stars were additionally supported by ≥0.95 posterior probability in Bayesian analysis. Bootstrap supports <70% in ML analysis were excluded unless nodes were supported by Bayesian analysis. Tips with black triangles were taxa that successfully reproduced upon exposure to *Leptocybe* sp. lineage B gall under laboratory conditions (except *Megastigmus* sp. 5 tested and failed to reproduce). Outgroup was from genome of *B. dorsalis* (Bunnefeld et al., 2018). Reference sequences were obtained from Auger‐Rozenberg et al. (2006) (suffix A. M.) and Roques et al., 2016 (suffix R. A.)

PHYML inference based on the 28S rDNA, applying the Jmodeltest2‐suggested model (TPM1 + I + G) constructed a phylogenetic tree with 30 taxa including the outgroup with log‐likelihood −2861 (Figure 3a). The species with metallic color from Australia, identified as *Bootanomyia* (Doğanlar, 2011), formed a highly supported group (98% bootstrap) and nested inside other *Megastigmus* taxa. The *Megastigmus* species were considerably divergent with some taxa forming well‐supported groups, although most associations had bootstrap support values below 50% (hereby referred as unsupported) or from 50% to less than 70% (weakly supported). Two clades of well‐supported *Leptocybe* associates were the *M. lawsoni* complex (91%) and the trio group of *M. zvimendeli*, *M. pretorianensis,* and *M. manonae* (100%), *Megastigmus zebrinus* presence in Australia has not been confirmed with molecular evidence in our data. The 28S rDNA sequences of *M. zebrinus* from South Africa and Vietnam were identical and were placed closest to the *M. lawsoni* complex (unsupported). *Megastigmus* sp. 1, associated with *Leptocybe* sp. lineage B and *Ophelimus* galls in various location, was placed close to a large, black species with a long ovipositor (*Megastigmus* sp. 5, 68% support value). The ability of reproducing upon exposure to *Leptocybe* sp. lineage B galls in the laboratory was not restricted to any clade (Figure 3). Such capacity was even recorded for the species *Bootanomyia* sp. 2, which fell well within the *Bootanomyia* group. Regarding the phytophagous taxa, all species were placed in an unsupported group separated from the eucalypt *Megastigmus*. The speciation nodes were separated by various branch lengths. However, inference of speciation time and factors influencing evolution rates was not possible with the limited taxon sampling and markers.

The main clades supported by 28S DNA persisted in the tree reconstructed from the concatenated dataset (Figure 3b). Bootstrap support for the Australian *Bootanomyia* species, the *M. lawsoni* species complex, and the trio (*M. zvimendeli, M. pretorianensis, M. manonae*) were 98%, 100%, and 100%, respectively. In contrast, the phytophagous taxa *M. pistaciae*, *M. grewianae,* and M. *helinae* were nested inside three different clades (unsupported). ML and BA results were generally in congruence, except a clade containing *M. manonae* and *M. pretorianensis* received 96% support in the concatenated dataset in ML but was neither supported by BA of the same dataset (Figure 3b) nor any of the 28S trees (Figure 3a).

In the COI phylogeny (Appendix S5), most of the interspecies clades received very low support, except for the lawsoni group (79% bootstrap, posterior probability 1), the pair *M. pretorianensis*/*M. manonae* (83%, 1), and the *Megastigmus* sp. 9/*Megastigmus* sp. 6 (87%, 1). The four eucalypt‐associated *Bootanomyia* species were placed in two different clades in the ML tree but grouped into one clade in BA tree, and *M. zvimendeli* was placed closer to the bulk of the *Bootanomyia* species. The topology of the COI tree differed greatly between ML and BA, mainly because unsupported ML clades obtained <50% posterior probability support in BA (Appendix S5).

Appendix S6 provides a phylogeny based on a 631 bp 28S alignment of a larger number of taxa with additional Megastigmidae sequences from previous authors (Auger‐Rozenberg et al., 2006; Janšta et al., 2018; Roques et al., 2016). Like Janšta et al. (2018), the Paleartic *Bootanomyia* species were placed in a sister group to the Australian megastigmids. However, sequences from the genera *Paramegastigmus*, *Malostigmus*, *Bortesia,* and *Neomegastigmus* and Australian taxa of *Bootanomyia* were nested inside the taxa from *Megastigmus*.

## 4 DISCUSSION

### 4.1 Molecular markers delimited *Megastigmus* and confirmed species associated with eucalypt galls

Except *M. zvimendeli* (discussed in Le et al., 2020) and *M. zebrinus* (discussed herein), the COI sequences obtained from eucalypt galls did not match any published sequence of phytophagous *Megastigmus*. The number of species and assignment of sequences to species was consistent for the two species delimitation methods GMYC and ABGD. Eucalypt‐associated species are separated from each other by a clear barcoding gap between the maximum intraspecies distance of 1.8% and the minimum interspecies distance of 5.6% (KP80 distance, Appendix S3). However, that barcode gap was not applicable for the phytophagous group. Several pairs of phytophagous species (Boivin et al., 2014) were recorded with distances of 2.6%, 2.5%, and even as low as 1.7%. This can partly be explained by the higher divergence in mtDNA of parasitic wasps than in nonparasitic wasps (Castro, Austin, & Dowton, 2002; Dowton & Austin, 1995). Figure 1 further illustrates the distribution pattern of pairwise distances between species associated with eucalypt galls (as presumptive parasitic/entomophagous *Megastigmus*) and between phytophagous *Megastigmus* species.

Among the species groups suggested by the phylogenetic analysis, a trio including *M. zvimendeli*, *M. pretorianensis,* and *M. manonae* is of interest. The first species (*M. zvimendeli*) commonly associates with *Leptocybe* sp. lineage B in Queensland and northern New South Wales, Australia. This species has been released as a biocontrol agent against *Leptocybe* spp. in Israel (Mendel et al., 2017), and our recent study revealed the establishment of this species in Israel, Kenya, China, and India (Le et al., 2020). *Megastigmus pretorianensis* was first described from South Africa as a *Leptocybe* spp. gall associate. In Australia, the species was recorded in association with a local *Leptocybe* sp. in a single location in Jindabyne, NSW. *Megastigmus manonae* specimens were found from galls of *Leptocybe* sp. lineage B and small blister galls on eucalypt leaves. These three species are small (ca. 1–1.2 mm excluding head and ovipositor) and have clavate antenna and two pairs of scutellar setae with the second pair near the rear end of the scutellum. Further research into host specificity and life history traits of these species is expected to contribute to their use as *Leptocybe* spp. natural enemies.

*Megastigmus lawsoni*, established in Israel as a *Leptocybe* spp. biocontrol agent (Mendel et al., 2017), represents at least three and likely four cryptic species differing in both COI mtDNA and 28S rDNA sequences. Female specimens of this group bear a single pair of setae on the scutellum, and male specimens have a distinct black patch around the median part of the transscutal articulation. We were unable to find reliable morphological characters assisting delimitation of species in this group and therefore treat *M. lawsoni* as a species complex. In our collection, the most common species (tentatively referred to as *M. lawsoni* 4) was characterized by a COI pseudogene differing from the functional genes by one deletion mutation. COI marker can be used to identify the exact species established in Israel and consequently correctly link biocontrol profile with identity of this species.

Our phylogeny placed *M. zebrinus* within the bulk of other eucalypt‐associated species, but the position of this species was unsupported and phylogeny was unresolved. In Roques et al. (2016), the position of *M. zebrinus* relative to phytophagous *Megastigmus* was also unsatisfactorily established. Like *M. zebrinus*, positions of the phytophagous taxa have not been resolved. The 28S‐based phylogeny lacks resolution at deeper nodes (Figure 3a), and none of these nodes were further clarified by concatenation of COI and 28S dataset (Figure 3b).

In the study, the COI‐based species delimitation results were confirmed by a unique 28S sequence for each species and were therefore unlikely to be misinterpreted. However, the phylogeny inferred from COI data was unstable and highly unresolved, which can be explained by the saturation of COI sequences and possible violation of orthology rule. Saturation, indicated by the very low transition/transversion ratio (*T_i_*/*T_v_* = 0.50, plotted in Appendix S7), occurred when the sequences undergo excessive mutations so that the estimation of mutational changes is no longer accurate and the sequences lost their phylogenetic values (Duchene, Ho, & Holmes, 2015; Purvis & Lindell, 1997; Yang & Yoder, 1999). Furthermore, violation of orthology, which is a factor contributing to phylogenetic incongruence (Ballard & Whitlock, 2004; Bensasson, Zhang, Hartl, & Hewitt, 2001; Som, 2014), cannot be excluded. In the study, pseudogenes have been found for *M. zvimendeli* and *M. lawsoni* 4. For *M. zvimendeli,* even with the alternative primer pair, high‐quality reading results were only obtained with the reverse primer, suggesting the presence of a second unknown PCR product. *Megastigmus manonae* haplotypes 4 and 5 were found with double peaks in several sites, which suggested the coexistence of different mitochondrial copies of the same gene. Based on the mutational saturation and the discussed complex evolution history of COI DNA, at this stage we relied on the 28S dataset in the inference of phylogenetic relationships.

Nevertheless, our results confirmed the paraphyletic status of *Bootanomyia,* in line with Janšta et al. (2018). The Australian species of *Bootanomyia* fell in a distinct clade with 98% support that separates itself from the outgroup taxon (*B. dorsalis*) and is nested inside the studied *Megastigmus* taxa. In contrast, the monophyly of *Megastigmus* was unsupported in the concatenated dataset and rejected in the 28S tree by the Australian *Bootanomyia* nesting inside *Megastigmus* taxa. As the family Megastigmidae has an Australian common ancestor (Janšta et al., 2018) and *Megastigmus* is the largest genus in the family, the possibility of a paraphyletic *Megastigmus* cannot be ruled out. In Janšta et al. (2018), *Megastigmus* was supported in ML and Bayesian analysis but not supported in Maximum parsimony analysis. The relationships among *Megastigmus*, *Malostigmus,* and *Neomegastigmus* were differently inferred in three analyses (Janšta et al., 2018). Askew et al. (2013) believed the use of the genus name *Bootanomyia* lead to paraphyletic status of *Megastigmus*. With genome sequencing technology, complete mitochondrial genome (Lee, Choi, Kim, Jeon, & Kim, 2018) and Ultra‐Conserved Elements (Cruaud et al., 2019) have become accessible for phylogenetic study. An investigation using these tools at family level is expected to fully investigate the evolution history of COI and phylogeny of *Megastigmus*/Megastigmidae.

### 4.2 *Megastigmus zebrinus* identity, a case study

*Megastigmus zebrinus* was described as a gall‐former from South Africa and Australia associated with fruits of *Syzygium cordatum* and *E. camaldulensis*, respectively (Grissell, 2006). It was later recorded to associate with *Leptocybe* galls from Thailand (Doğanlar & Hassan, 2010), Argentina (Hernández, Aquino, Cuello, Andorno, & Botto, 2015), and South Africa, where its status was reclassified from primary galler to probable parasitoid (Klein, Hoffmann, Neser, & Dittrich‐Schröder, 2015). Our molecular data confirm the occurrence of *M. zebrinus* in South Africa (*M. zebrinus* haplotype 1 and 2) and recorded its presence in Vietnam (*M. zebrinus* haplotype 3) and Israel. However, we were unable to obtain *M. zebrinus* specimens from Thailand and Argentina and failed to find the species in Australia. With a record of association with a noneucalypt host plant and a multicontinental distribution, *M. zebrinus* could be the model insect for an origin tracing study using mitochondrial marker, like Scheffer and Grissell (2003), and for understanding the host‐shifting process in *Megastigmus*.

Specimens of *M. zebrinus* from Israel were strongly discolored, and their DNA was damaged during preservation. Amplification of the desired long segment of COI mtDNA and 28S rDNA failed for these specimens, but a short fragment (190 bp excluding primer binding sites) was amplified using the internal primer pair 2222‐COI‐F/2413‐COI‐R. These DNA fragments were aligned and were identical to the matching region of *M. zebrinus* haplotype 1. We herein argue for the occurrence of *M. zebrinus* in Israel based on this evidence:

- The PHYML‐based tree built from the obtained 190 bp COI fragment separated this species well from others (Appendix S8). In identification of parasitic Hymenoptera, diagnoses have been possible with COI fragments of as short as <150 bp (Andersen & Mills, 2012).
- For DNA barcoding, misidentification risk may result from the presence of a pseudogene that preferentially amplify over the target barcode gene (Bensasson et al., 2001; Song, Buhay, Whiting, & Crandall, 2008). It is unlikely that a pseudogene was the case here as the sequence chromatograms were free of double peaks, indicating the presence of a single PCR product. No disruption to the amino acid sequence was observed, suggesting that the gene is coding for protein and functional.
- Regarding morphology, specimens from Israel displayed the important characters found in South African *M. zebrinus*: two interocellar setae close to the midocellus (Grissell, 2006), scutellum bearing three pairs of black, conspicuous scutellar setae approximately at equidistance. Compared to dry paratypes of *M. zebrinus* (ANIC111470, ANIC111471), the alcohol preserved specimens have a lower *pol.l*:*ool.l* ratio (2.8–3.1 vs. 3.3–3.4), but this may have resulted from preservation condition rather than true morphological differences.

Another species, *M. leptocybus,* was described from the same locality that the Israeli *M. zebrinus* was collected (Doğanlar & Hassan, 2010). In the original description, *M. leptocybus* was distinguished from other species by having the pedicel plus flagellum shorter (0.8×) than the width of the head (Doğanlar & Hassan, 2010). The specimens identified herein as *M. zebrinus* have *pdl.flg*/*head.b* = 1.2 – 1.3 (measured on seven alcohol preserved individuals) and hence could not be keyed out to *M. leptocybus*. However, when a further attempt was made to examine the available paratypes of *M. leptocybus* in the Australian National Insect Collection (ANIC) (ANIC 111467, 2 females on a card) and *M. zebrinus* (ANIC 111470, ANIC 111471, and multiple paratypes deposited at Queensland Museum (QM)), we failed to find clear morphological characters to distinguish these species and did not observe a low *pdl.flg*/*head.b* ratio in *M. leptocybus*. We therefore suspect *M. zebrinus* and *M. leptocybus* are synonyms but this requires molecular confirmation.

Photographic illustration of *M. zebrinus* from South Africa and Israel, and the ANIC paratypes of the Israeli *M. leptocybus* are provided (Appendix S9).

### 4.3 Molecular work is required to overcome the uncertainty of *Megastigmus* taxonomy

Morphological data assisted in matching the species recognized in this study with those described in multiple works of Girault (Girault, 1915, 1925, 1929) and redescribed by Doğanlar and Hassan (2010). For example, the original description of *M. eucalypti* (Girault, 1915) described a species with “flagellum black, funicle 1 a little shorter than the pedicel, 1 somewhat longer than wide. Distal funicle joint a little wider than long. Sometimes, the propodeum is wholly black. Head lemon‐yellow” and females with “length, 2.25 mm., exclusive of ovipositor which is extruded for a length somewhat over half that of the body.” These were important characters observed in the large form of *Megastigmus* sp. 1. The smaller form of *Megastigmus* sp. 1 is similar to the QM specimens identified as *M. fieldingi* by Grissell (2006) in shape, size, and body color. The scutellar setae form “3b” in *Megastigmus* sp. 6 and *Megastigmus* sp. 8 (Table 2) was an important character, “second setae of the scutellum twice closer to 3 than to 1,” in the original description of *M. amamoori* (Girault, 1925) and *M. pallidiocellus* (Girault, 1929) (QM holotypes T5011 and T5021). However, for females of most species, the color and shape of collar and propodeum and visibility of mesoscutal pattern appeared to change when body size and color varied. Identification to established names was therefore confounded by the high variation in size and color of species in our study, and the poor condition of Girault's type specimens.

Bouček (1988) suggested that a revisional study would lead to the description of many new species and synonymies of current described cases in Australia. Protasov, Doĝanlar, La Salle, and Mendel (2008) also highlighted the need of a detailed revision of the European fauna in identifying an Israeli local species (likely *M. leptocybus*, although not explicitly stated in the literature, see reasoning in Le et al., 2018). Several morphological characters, such as the number and arrangement of scutellar setae (Doğanlar & Hassan, 2010), were found to assist species delimitation. Despite that, we believe that future revision needs to involve molecular work on type specimens or specimens collected from the type localities and historical hosts, and association of obtained sequences with designated type specimens. Noninvasive DNA extraction techniques have been proved to successfully extract DNA fragments for species identification of parasitic Hymenoptera after up to 100 years preservation (Andersen & Mills, 2012). The pending case of *M. leptocybus*, as discussed in the previous section, could only be resolved with certainty using supplementary molecular data and a worldwide collaboration of *Megastigmus* researchers. Molecular work can also be applied for many species recently reported to associate with invasive *Leptocybe* spp. in different parts of the world (e.g., *M. thailandiensis* and *M. thitipornae* in Thailand, *M. dharwadicus* in India, *M. brasiliensis* in Brazil, and *M. zebrinus* in Argentina), all of which we were unable to source for our study despite extensive collections in Thailand (see Le et al., 2018). Our recent attempt confirming the synonymies of *M. zvimendeli*, *M. sichuanensis,* and *M. icipeensis* (Le et al., 2020) could set an example of this approach.

Despite the discussed limitations, our study has successfully contributed to the understanding of species composition and species delimitation for eucalypt‐associated *Megastigmus*. The constructed phylogeny identified several species groups of importance to *Leptocybe* spp. biocontrol. Data of COI mtDNA sequences clearly delimited species and can be further applied in designing diagnostic primer for use in monitoring of *Leptocybe* spp. biocontrol programs, while the presented morphological characters form a new baseline in understanding the morphological variation within species and between eucalypt gall‐associated *Megastigmus*.

## Conflict of Interest

The authors declared that there is no financial, general, and institutional competing interests regarding the publication of this article.

## AUTHOR CONTRIBUTION

**Ngoc Hoan Le:** Conceptualization (equal); Data curation (lead); Formal analysis (equal); Investigation (equal); Methodology (equal); Project administration (equal); Writing‐original draft (equal); Writing‐review & editing (equal). **Helen F Nahrung:** Conceptualization (equal); Formal analysis (equal); Funding acquisition (equal); Investigation (equal); Supervision (equal); Validation (equal); Writing‐original draft (equal); Writing‐review & editing (equal). **Jess Morgan:** Conceptualization (equal); Formal analysis (equal); Investigation (equal); Methodology (equal); Supervision (equal); Writing‐original draft (equal); Writing‐review & editing (equal). **Steven Ogbourne:** Conceptualization (equal); Methodology (equal); Software (equal); Writing‐review & editing (equal). **Simon Lawson:** Conceptualization (equal); Formal analysis (equal); Funding acquisition (lead); Methodology (equal); Project administration (equal); Resources (lead); Supervision (lead); Writing‐original draft (equal); Writing‐review & editing (equal).

## Supporting information

Appendix S1

Appendix S2

Appendix S3

Appendix S4

Appendix S5

Appendix S6

Appendix S7

Appendix S8

Appendix S9

## Data Availability

DNA sequences generated in this study have been submitted to the GenBank databases under accession number MT375387 ‐ 395, MT380065 ‐ 101, MT383677 ‐ 740 and MN165877 ‐ 951 (detailed in Appendix S2). Data for the reproduction of the results in this paper have been uploaded to The Open Science Framework and are accessible via this link: https://osf.io/nqbmw/?view_only=08c72f1ff3244ec7b8a01e7df1ba633e. File 1. Sequence alignment, fasta, COI sequence alignment for GMYC analysis. File 2. Sequence alignment, fasta, 865 bp 28S sequence alignment for phylogenetic reconstruction. File 3. Sequence alignment, fasta, COI sequence alignment for phylogenetic reconstruction. File 4. Sequence alignment, fasta, concatenated sequence alignment for phylogenetic reconstruction. File 5. Sequence alignment, fasta, 631 bp 28S sequence alignment for phylogenetic reconstruction. File 6. Log files, compressed (zip), tree reconstruction using BEAST 2.6.3 as input for GMYC. File 7. Log files, compressed (zip), analysis of 28S alignment with MrBayes 3.2.7. File 8. Log files, compressed (zip), analysis of concatenated alignment with MrBayes 3.2.7. File 9. Log files, compressed (zip), analysis of COI alignment with MrBayes 3.2.7
