# Supplementary material for: Molecular markers reveal diversity in composition of Megastigmus (Hymenoptera: Megastigmidae) from eucalypt galls
Source: Ecol Evol. 2020 Sep 25;10(20):11565–78. doi: 10.1002/ece3.6791 (PMC7593149; doi:10.1002/ece3.6791)
Supplement: Supplementary file 1 — Appendix S1 [file ECE3-10-11565-s001.docx]

| **Supplementary document 1.** Gall morphology and coemergence of *Megastigmus* species | | | | | | |
| --- | --- | --- | --- | --- | --- | --- |
| **No.** | **Location, Longitude/latitude** | **Gall type/Eucalypt plant** | **Megastigmidae species** | **Other associates** | **Dominant emergee** | **Illustration** |
| 1 | ACT, Symonston  (-35.3527, 149.1421) | Axial bud  *E. blakelyi* | *Megastigmus* sp. 5 | Cecidomyiidae Encyrtidae Pteromalidae *Cirrospilus* sp. | *Ophelimus* sp. | 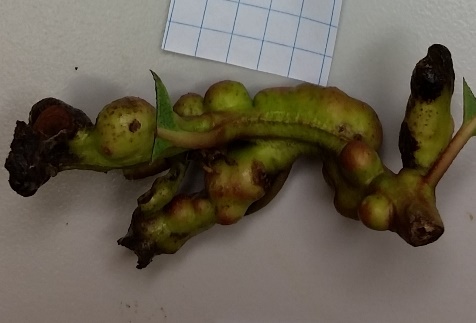 |
| 3 | NSW, Cassilis  (-32.0536, 149.9333) | Leaf | *Megastigmus* sp. 1 + *Bootanomyia* sp. | Unidentified Eulophidae  *Ophelimus* sp. | - | 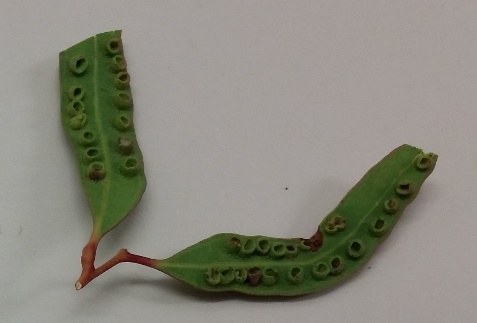 |
| 4 | NSW, Cassilis  (-32.0536, 149.9333) | Terminal bud | *Megastigmus* sp. 4 + *Megastigmus* sp. 8 | Cecidomyiidae  *Ophelimus* sp. | - | 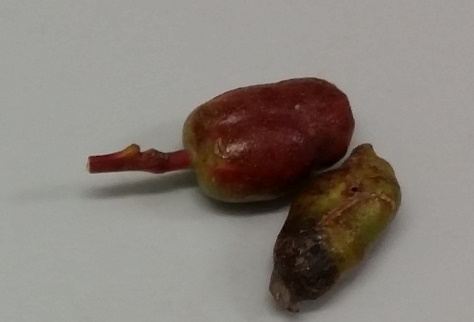 |
| 5 | NSW, Run-o-waters  (-34.7835, 149.6617) | Stem  *Eucalyptus* sp. | *Bootanomyia* sp. 4 | Encyrtidae,  Eurytomidae,  Pteromalidae  *Ophelimus* sp. | Unidentified Eurytomidae | 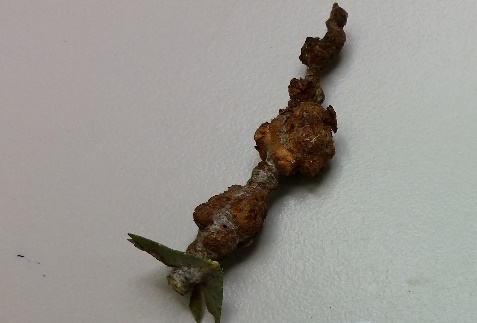 |
| 6 | NSW, Shannons Flat  (-35.9043, 148.9669) | Leaf  *Eucalyptus* sp. | *Megastigmus* sp. 5 | *Ophelimus* sp. | - | 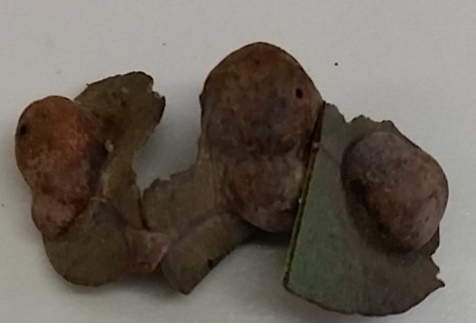 |
|  | NSW, Shannons Flat  (-35.9043, 148.9669) | Leaf  *Eucalyptus* sp. | *Megastigmus* sp. 9 | *Ophelimus* sp. | - | 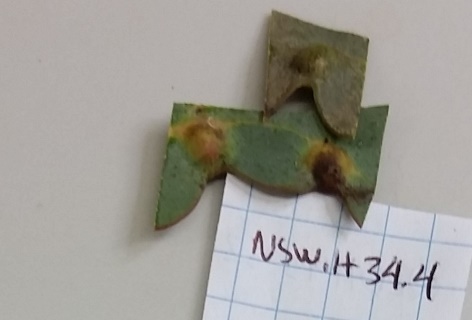 |
| 8 | QLD, Canina  (-26.1239, 152.7418) | Leaf  *Eucalyptus* sp. | *Megastigmus* sp. 1 | *Cirrospilus* sp.  *Ophelimus* sp. | *Cirrospilus* sp. | 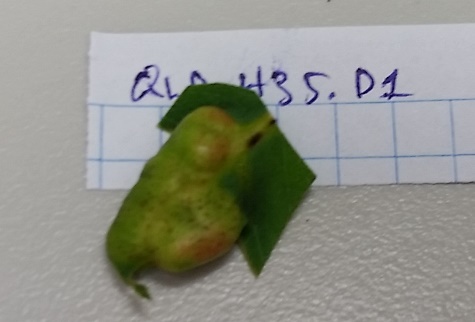 |
| 9 | QLD, Coondoo  (-26.1958, 152.9216) | Terminal bud  *Eucalyptus* sp. | *Megastigmus* sp. 1 | Cecidomyiidae,  Encyrtidae,  Eurytomidae *Cirrospilus* sp.,  Unidentified Eulophidae | *Ophelimus* sp. | 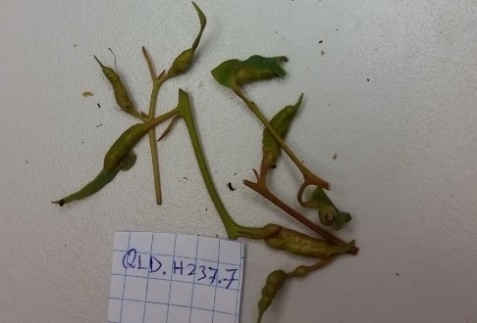 |
| 10 | QLD, Federal  (-26.4251, 152.8082) | Leaf  *Eucalyptus* sp. | *Megastigmus manonae* | *Selitrichodes kryceri Selitrichodes neseri* Unidentified Eulophidae  *Ophelimus* sp. |  | 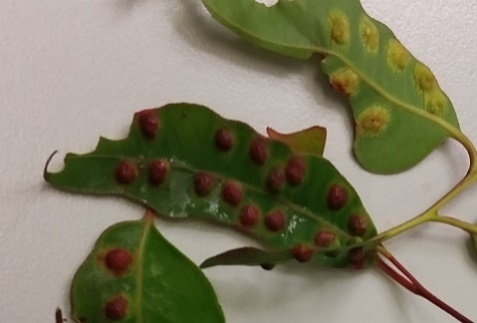 |
| 11 | QLD, Fernvale  (-27.4876, 152.6629) | Leaf  *Eucalyptus* sp. | *Megastigmus* sp. 1 | Eupelmidae *Closterocerus* sp. | *Ophelimus* sp. | 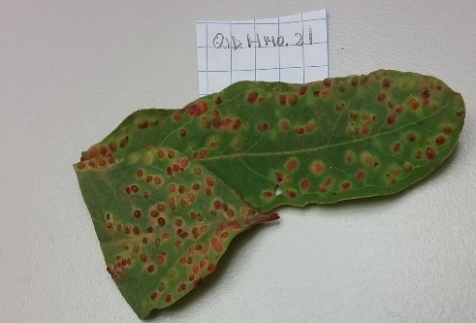 |
| 12 | QLD, Lawes  (-27.5583, 152.3341) | Leaf  *Eucalyptus* sp. | *Megastigmus* sp. 1 + *Megastigmus* sp. 4 | Encyrtidae,  Pteromalidae  *Ophelimus* sp. | Unidentified Pteromalidae | 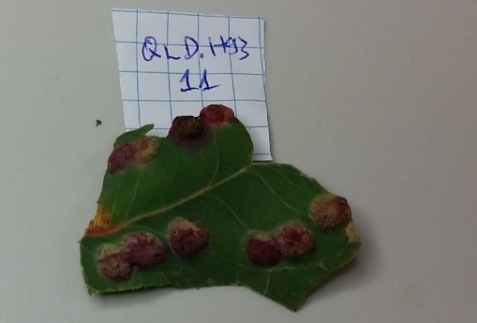 |
| 13 | QLD, Maroochydore  (-26.7190, 153.0631) | Stem  *Eucalyptus* *robusta*. | *Megastigmus* sp. 2 | *Cirrospilus* sp.  *Ophelimus* sp. | - | Not available |
| 14 | QLD, Rosenthal Heights  (-28.2342, 151.9812) | Axial bud  *Eucalyptus* *cinerea*. | *Megastigmus* sp. 5 | *Ophelimus* sp. | - | Not available |
| 15 | QLD, Yarraman  (-26.8516, 151.9676) | Leaf  *Eucalyptus* sp. | *Megastigmus* sp. 11 | *Cirrospilus* sp.  *Ophelimus* sp. | *Cirrospilus* sp. | 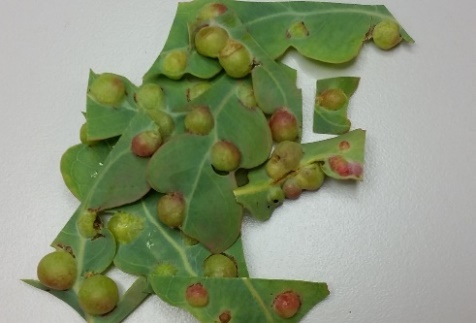 |
| 16 | VIC, Noorinbee  (-37.6650, 148.8020) | Leaf  *Eucalyptus* sp. | *M. manonae* | *Quadrastichus* sp.  unidentified Tetrastichinae, Encyrtidae, Eurytomidae, Mymaridae | *Leptocybe* sp.  (local) | 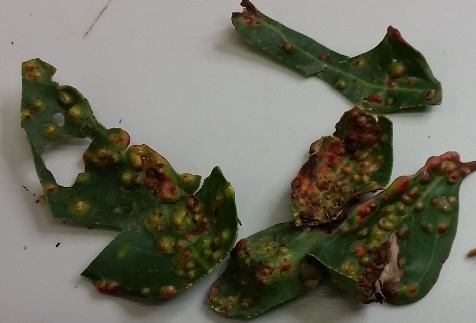 |
| 17 | NSW, Jindabyne  (-36.4143, 148.6249) | Leaf  *Eucalyptus* sp. | *M. pretorianensis*  *Megastigmus* sp. 6 | *Selitrichodes* sp.  Unidentified Eulophidae, Mymaridae | *Leptocybe* sp. (local, confirmed with DNA barcode) | 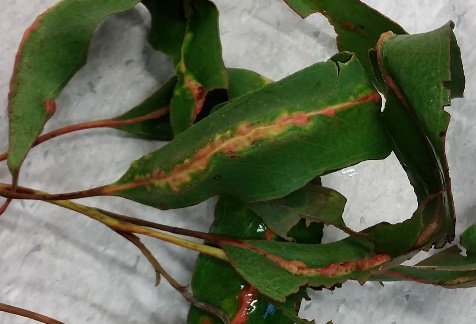 |
| 18 | QLD, Miva  (-5.9439, 152.5076) | *E. tereticornis* | *M. zvimendeli* | *Q. mendeli*  Unidentified Cecydomyiidae | *Leptocybe* sp. lineage B | 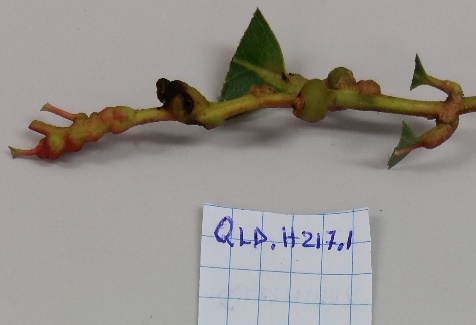 |
| 19 | QLD, Miva  (-5.9439, 152.5076) | *E. tereticornis* | *Megastigmus* sp. 1 | *Q. mendeli*  *S. neseri*  *S. kryceri*  *Unknown* Encyrtidae  Cecydomyiidae | *Leptocybe* sp. lineage B | 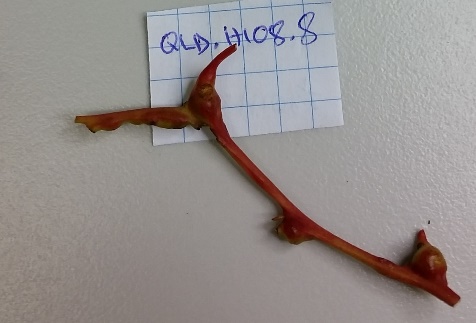 |
| 20 | NSW, Fernside  (-28.7941, 153.1949) | E. camal/ tereticornis | *M. lawsoni* 4  *Megastigmus* sp. 1 | *Q. mendeli*  *S. kryceri*  *Ophelimus* sp.  Unknown Mymaridae | *Leptocybe* sp. lineage B | 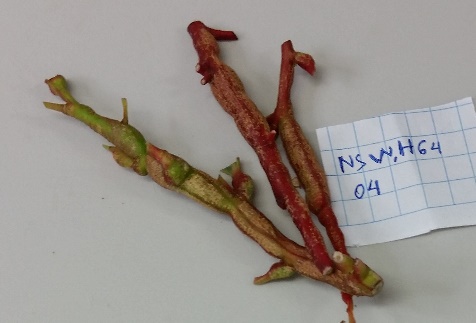 |

Note: although all identified plants belong to the genus *Eucalyptus*, collection included young seedlings from all three eucalypt genera *sensu lato*, *Eucalyptus*, *Corymbia* and *Angophora*.
