# Supplementary material for: Molecular markers reveal diversity in composition of Megastigmus (Hymenoptera: Megastigmidae) from eucalypt galls
Source: Ecol Evol. 2020 Sep 25;10(20):11565–78. doi: 10.1002/ece3.6791 (PMC7593149; doi:10.1002/ece3.6791)
Supplement: Supplementary file 3 — Appendix S3 [file ECE3-10-11565-s003.docx]

| **Supplementary Document 3.** KP80 pairwise distance of COI sequences (lower) and proportion of pairwise differences of 28S rDNA (upper) between *Megastigmus* species associated with eucalypt galls. Number in parentheses denote COI haplotype number for each species. For species with more than one unique COI sequence, distance values were the average number and values in diagonal cells denote intra-species KP80 distance (minimum and maximum value, if applicable). Calculation was based on aligned 849bp for COI mtDNA and aligned 858bp for 28S rDNA. Alignment gaps (for 28S DNA) and ambiguous bases (for COI) were removed from pairwise calculation. | | | | | | | | | | | | | | | | | | | | |
| --- | --- | --- | --- | --- | --- | --- | --- | --- | --- | --- | --- | --- | --- | --- | --- | --- | --- | --- | --- | --- |
|  | **1** | **2** | **3** | **4** | **5** | **6** | **7** | **8** | **9** | **10** | **11** | **12** | **13** | **14** | **15** | **16** | **17** | **18** | **19** | **20** |
| 1. *Bootanomyia* sp. 1 | - | 0.020 | 0.022 | 0.019 | 0.043 | 0.025 | 0.026 | 0.030 | 0.027 | 0.025 | 0.027 | 0.043 | 0.040 | 0.033 | 0.032 | 0.034 | 0.040 | 0.040 | 0.043 | 0.032 |
| 2. *Bootanomyia* sp. 2 | 0.094 | - | 0.020 | 0.012 | 0.046 | 0.023 | 0.025 | 0.030 | 0.025 | 0.023 | 0.025 | 0.042 | 0.033 | 0.032 | 0.030 | 0.030 | 0.035 | 0.036 | 0.039 | 0.029 |
| 3. *Bootanomyia* sp. 3 | 0.112 | 0.107 | - | 0.019 | 0.046 | 0.023 | 0.025 | 0.032 | 0.027 | 0.026 | 0.028 | 0.044 | 0.036 | 0.033 | 0.032 | 0.033 | 0.041 | 0.042 | 0.044 | 0.033 |
| 4. *Bootanomyia* sp. 4 | 0.089 | 0.064 | 0.091 | - | 0.042 | 0.022 | 0.023 | 0.029 | 0.023 | 0.022 | 0.023 | 0.040 | 0.034 | 0.027 | 0.026 | 0.026 | 0.030 | 0.030 | 0.034 | 0.027 |
| 5. *Megastigmus* sp. 1 (3) | 0.095 | 0.091 | 0.116 | 0.082 | 0.001-0.002 | 0.026 | 0.027 | 0.022 | 0.028 | 0.028 | 0.028 | 0.041 | 0.034 | 0.037 | 0.036 | 0.036 | 0.042 | 0.042 | 0.044 | 0.035 |
| 6. *Megastigmus* sp. 2 (2) | 0.111 | 0.089 | 0.107 | 0.093 | 0.095 | 0.009 | 0.001 | 0.014 | 0.009 | 0.002 | 0.009 | 0.029 | 0.016 | 0.016 | 0.015 | 0.015 | 0.021 | 0.022 | 0.025 | 0.014 |
| 7. *Megastigmus* sp. 4 | 0.090 | 0.085 | 0.107 | 0.081 | 0.076 | 0.089 | - | 0.015 | 0.011 | 0.003 | 0.011 | 0.030 | 0.018 | 0.018 | 0.016 | 0.016 | 0.022 | 0.023 | 0.026 | 0.015 |
| 8. *Megastigmus* sp. 5 (3) | 0.102 | 0.103 | 0.115 | 0.094 | 0.090 | 0.089 | 0.097 | 0.004-0.012 | 0.014 | 0.015 | 0.016 | 0.035 | 0.021 | 0.025 | 0.023 | 0.023 | 0.028 | 0.028 | 0.028 | 0.021 |
| 9. *Megastigmus* sp. 6 (2) | 0.103 | 0.076 | 0.108 | 0.093 | 0.085 | 0.083 | 0.085 | 0.087 | 0.011 | 0.012 | 0.005 | 0.032 | 0.018 | 0.015 | 0.014 | 0.016 | 0.021 | 0.021 | 0.023 | 0.012 |
| 10. *Megastigmus* sp. 8 (2) | 0.093 | 0.079 | 0.102 | 0.086 | 0.078 | 0.085 | 0.078 | 0.103 | 0.077 | 0.002 | 0.012 | 0.029 | 0.019 | 0.019 | 0.018 | 0.018 | 0.021 | 0.022 | 0.023 | 0.016 |
| 11. *Megastigmus* sp. 9 | 0.106 | 0.085 | 0.123 | 0.099 | 0.102 | 0.098 | 0.090 | 0.110 | 0.056 | 0.093 | - | 0.032 | 0.020 | 0.015 | 0.014 | 0.016 | 0.021 | 0.021 | 0.026 | 0.012 |
| 12. *Megastigmus* sp. 10 | 0.097 | 0.107 | 0.120 | 0.099 | 0.102 | 0.109 | 0.081 | 0.109 | 0.106 | 0.086 | 0.113 | - | 0.034 | 0.032 | 0.030 | 0.030 | 0.040 | 0.042 | 0.044 | 0.034 |
| 13. *Megastigmus* sp. 11 | 0.094 | 0.069 | 0.112 | 0.085 | 0.077 | 0.085 | 0.078 | 0.089 | 0.077 | 0.087 | 0.093 | 0.110 | - | 0.027 | 0.027 | 0.027 | 0.030 | 0.034 | 0.035 | 0.025 |
| 14. *M. lawsoni* 1 | 0.105 | 0.103 | 0.128 | 0.106 | 0.102 | 0.099 | 0.094 | 0.094 | 0.090 | 0.097 | 0.108 | 0.113 | 0.101 | - | 0.001 | 0.006 | 0.025 | 0.025 | 0.027 | 0.015 |
| 15. *M. lawsoni* 2 | 0.095 | 0.101 | 0.123 | 0.102 | 0.099 | 0.091 | 0.087 | 0.089 | 0.073 | 0.094 | 0.095 | 0.112 | 0.094 | 0.066 | - | 0.005 | 0.023 | 0.023 | 0.026 | 0.014 |
| 16. *M. lawsoni* 3 | 0.114 | 0.087 | 0.114 | 0.094 | 0.090 | 0.095 | 0.081 | 0.089 | 0.078 | 0.097 | 0.098 | 0.110 | 0.089 | 0.078 | 0.060 | - | 0.026 | 0.026 | 0.028 | 0.016 |
| 17. *M. manonae* sp. n (6) | 0.094 | 0.081 | 0.095 | 0.088 | 0.086 | 0.083 | 0.086 | 0.084 | 0.069 | 0.064 | 0.081 | 0.087 | 0.087 | 0.090 | 0.089 | 0.082 | 0.004-0.018 | 0.006 | 0.009 | 0.026 |
| 18. *M. pretorianensis* (2) | 0.112 | 0.087 | 0.115 | 0.090 | 0.100 | 0.097 | 0.096 | 0.094 | 0.077 | 0.090 | 0.090 | 0.114 | 0.089 | 0.095 | 0.094 | 0.087 | 0.060 | 0.008 | 0.009 | 0.026 |
| 19. *M. zvimendeli* (4) | 0.096 | 0.091 | 0.119 | 0.089 | 0.101 | 0.108 | 0.092 | 0.111 | 0.091 | 0.086 | 0.100 | 0.110 | 0.096 | 0.110 | 0.102 | 0.105 | 0.081 | 0.080 | 0.001-0.004 | 0.028 |
| 20. *Megastigmus* *zebrinus* (3) | 0.108 | 0.095 | 0.113 | 0.089 | 0.099 | 0.091 | 0.085 | 0.104 | 0.086 | 0.089 | 0.095 | 0.114 | 0.094 | 0.094 | 0.079 | 0.079 | 0.083 | 0.092 | 0.105 | 0.005-0.013 |
