# Supplementary material for: Molecular markers reveal diversity in composition of Megastigmus (Hymenoptera: Megastigmidae) from eucalypt galls
Source: Ecol Evol. 2020 Sep 25;10(20):11565–78. doi: 10.1002/ece3.6791 (PMC7593149; doi:10.1002/ece3.6791)
Supplement: Supplementary file 4 — Appendix S4 [file ECE3-10-11565-s004.docx]

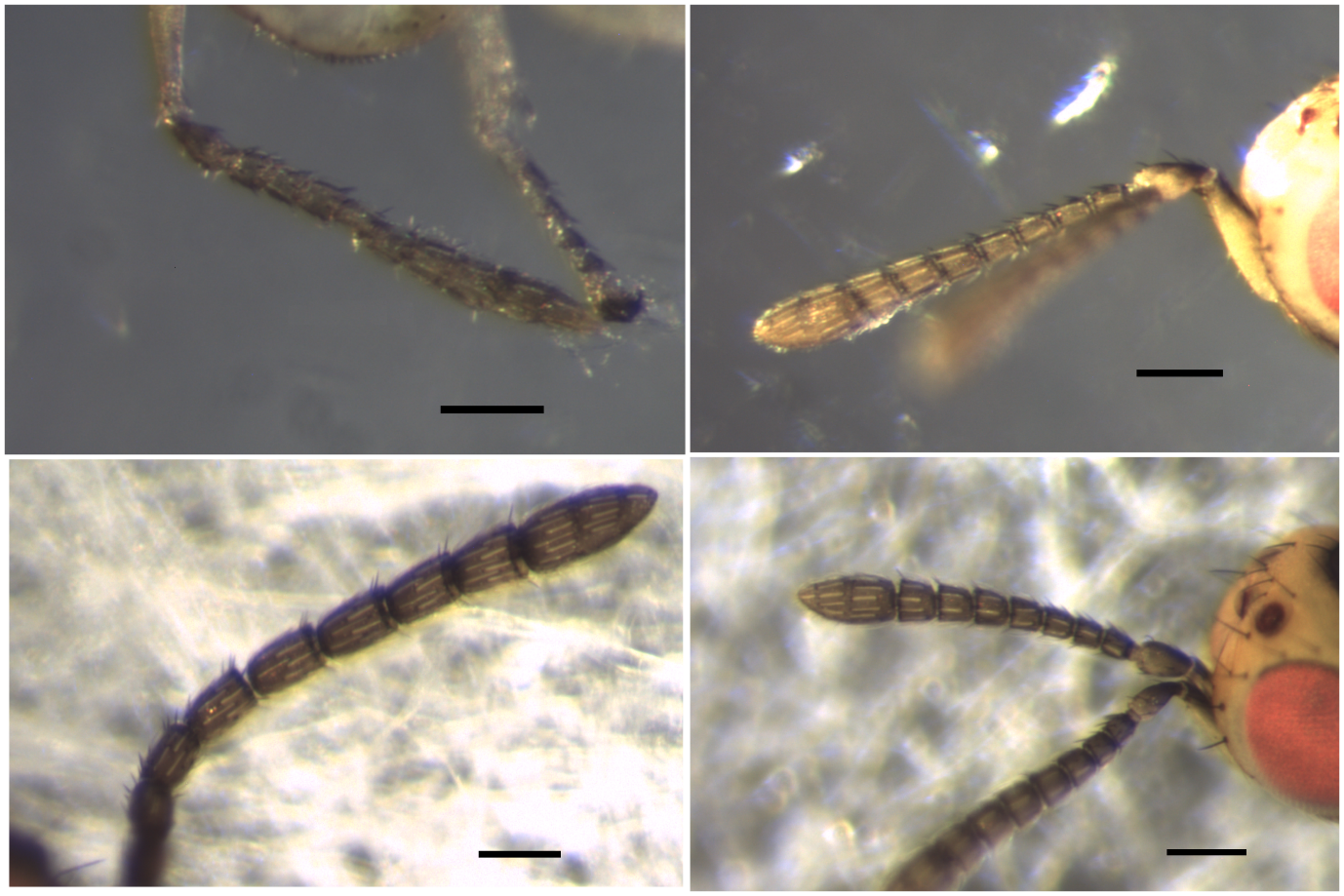


**Supplementary document 4.** Number of sensilla rows A. *Megastigmus* sp. 6 (1 row); B. *Megastigmus* sp. 2 (2 rows); C. *Megastigmus* sp. 1 (1 row); and D. *Megastigmus* sp. 10 (unclear). Scale bars approximate 0.1 mm
