# Supplementary material for: Molecular markers reveal diversity in composition of Megastigmus (Hymenoptera: Megastigmidae) from eucalypt galls
Source: Ecol Evol. 2020 Sep 25;10(20):11565–78. doi: 10.1002/ece3.6791 (PMC7593149; doi:10.1002/ece3.6791)
Supplement: Supplementary file 5 — Appendix S5 [file ECE3-10-11565-s005.docx]

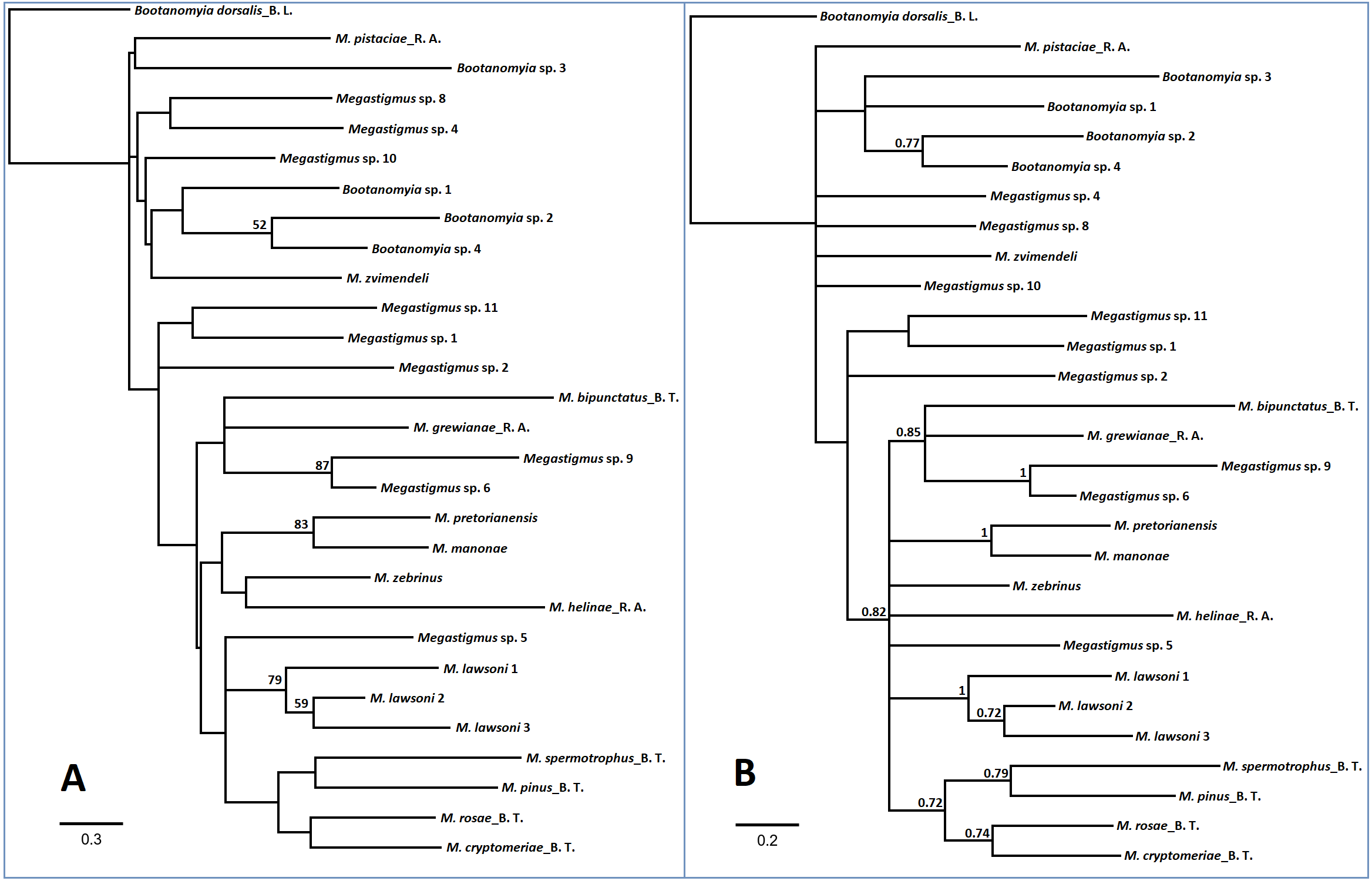


**Supplementary document 5**. Phylogeny of *Megastigmus* and *Bootanomyia* species based on COI mtDNA, 760 bp, using RAxML (A) and MrBayes (B), with model of evolution separately determined for three partitions (3 codon positions). Additional genbank entries were from Roques *et al.* (2016) (affix R. A.), Boivin *et al.* (2014) (affix B. T.). Outgroup was COI mtDNA from genome of *B. dorsalis* from Bunnefeld *et al.* (2018). Support values were shown for nodes with ≥ 50% bootstrap (for ML analysis) and ≥ 0.70 (for Bayesian analysis).
