# Supplementary material for: Molecular markers reveal diversity in composition of Megastigmus (Hymenoptera: Megastigmidae) from eucalypt galls
Source: Ecol Evol. 2020 Sep 25;10(20):11565–78. doi: 10.1002/ece3.6791 (PMC7593149; doi:10.1002/ece3.6791)
Supplement: Supplementary file 6 — Appendix S6 [file ECE3-10-11565-s006.docx]

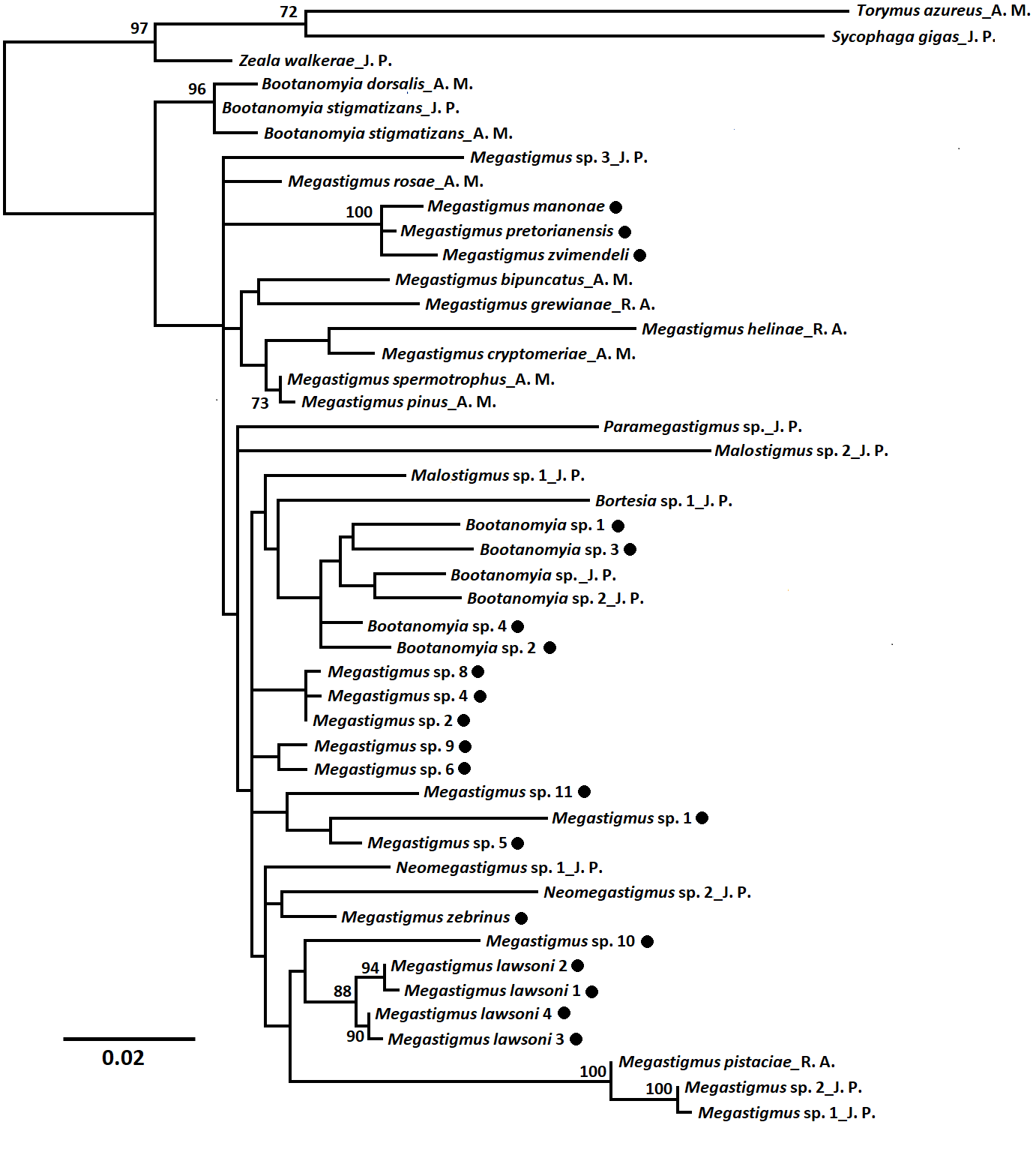


**Supplementary document 6**. Phylogeny of *Megastigmus* and *Bootanomyia* species based on 631 bp alignment of 28S rDNA and PHYML analysis. Model of evolution was SYM+I+G (Symetrical model with a proportion of invariable sites and Gamma-distributed among-site rate variation). Taxa with dots were from eucalypt galls extracted in the study. Additional genbank entries were from Auger‐Rozenberg *et al.* (2006) (affix A. M.), Janšta *et al.* (2018) (affix J. P.), Roques *et al.* (2016) (affix R. A.),. Outgroups were *Torymus aruzeus*, *Zeala Walkerae* and *Sycophaga gigas,* selected from Janšta *et al.* (2018).
