# Supplementary material for: Molecular markers reveal diversity in composition of Megastigmus (Hymenoptera: Megastigmidae) from eucalypt galls
Source: Ecol Evol. 2020 Sep 25;10(20):11565–78. doi: 10.1002/ece3.6791 (PMC7593149; doi:10.1002/ece3.6791)
Supplement: Supplementary file 7 — Appendix S7 [file ECE3-10-11565-s007.docx]

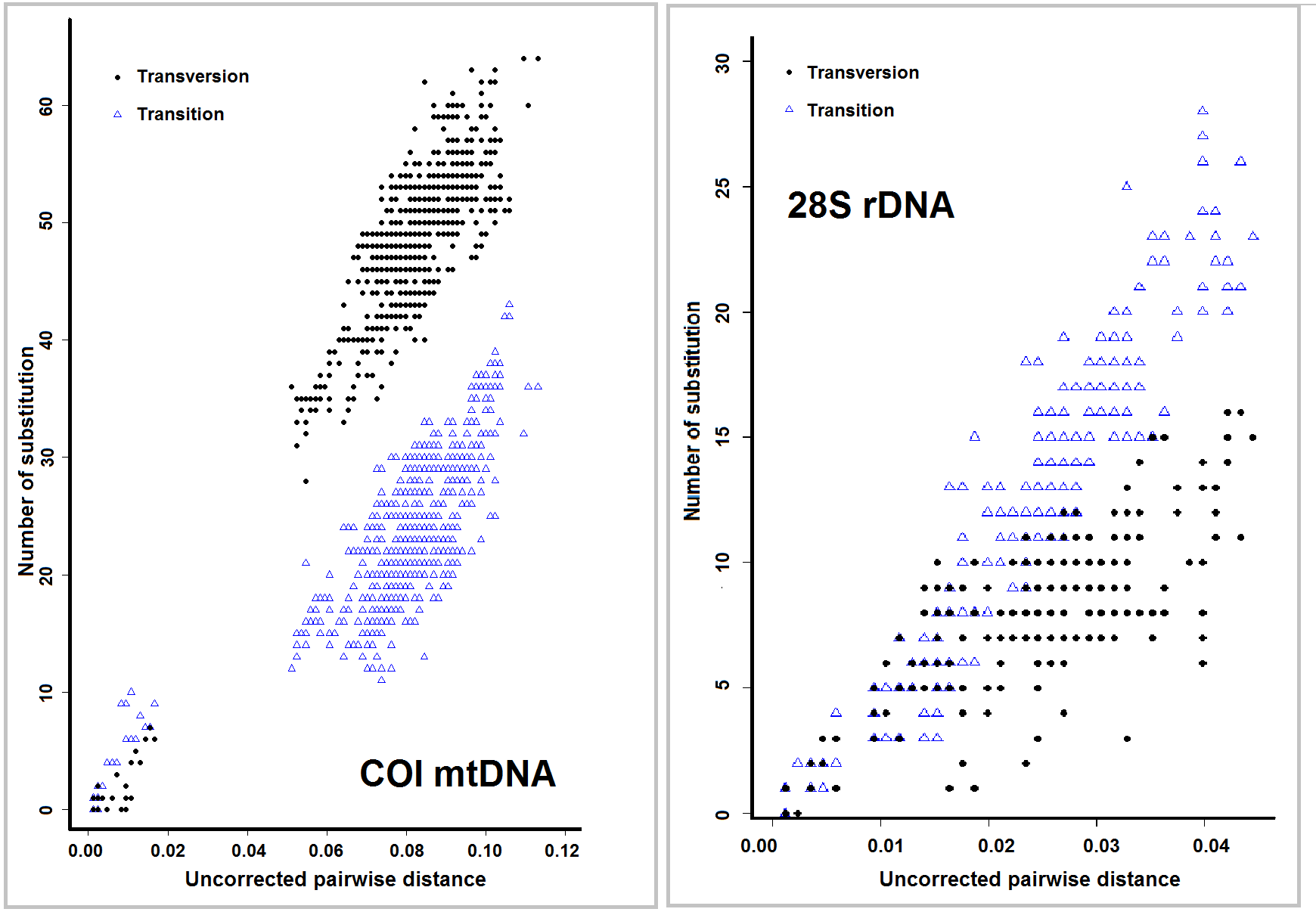


**Supplementary document 7***.*  Saturation plot illustrating number of transition and transversion in relation to uncorrected pairwise distances (proportion of base differences). Plots constructed using the trimmed alignment of unique sequences (38 sequences of 849bp for COI and 21 sequences of 858bp for 28S), on the R-based package R-based package SPIDER (Brown *et al.*, 2012).
