# Supplementary material for: Molecular markers reveal diversity in composition of Megastigmus (Hymenoptera: Megastigmidae) from eucalypt galls
Source: Ecol Evol. 2020 Sep 25;10(20):11565–78. doi: 10.1002/ece3.6791 (PMC7593149; doi:10.1002/ece3.6791)
Supplement: Supplementary file 8 — Appendix S8 [file ECE3-10-11565-s008.docx]

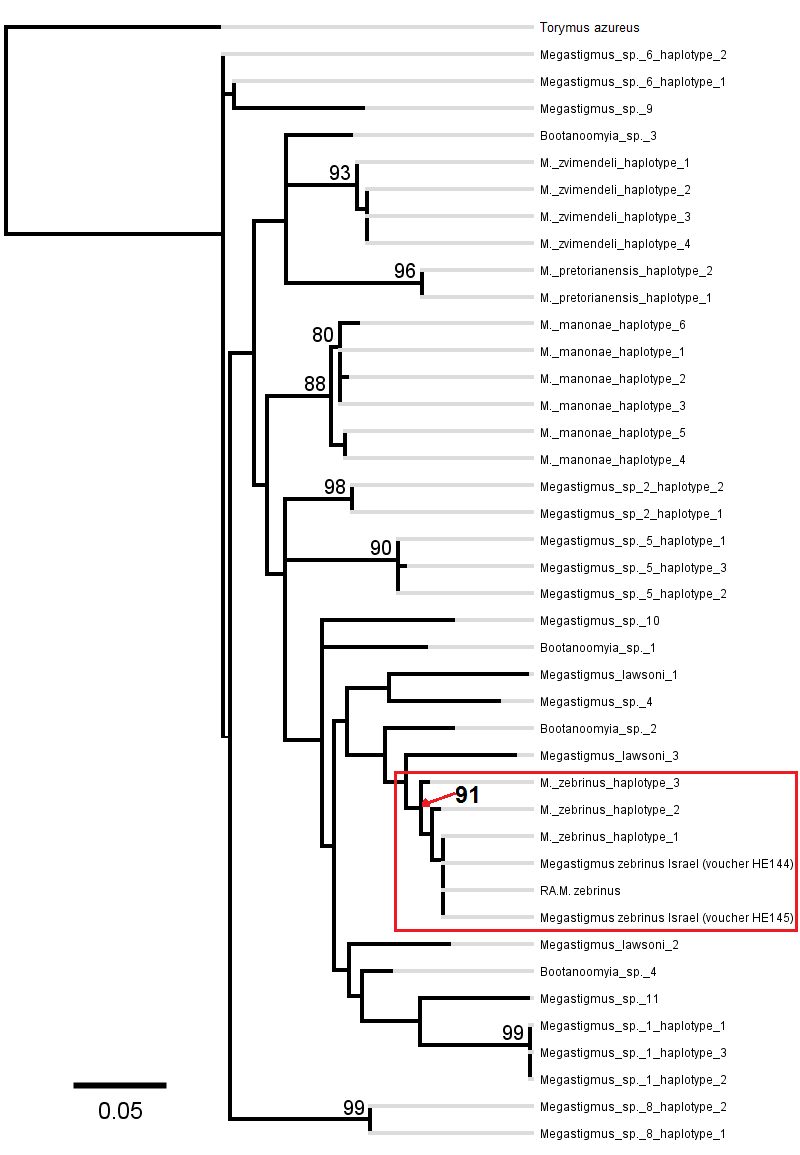


**Supplementary document 8.** PHYML phylogeny supporting identification of specimen voucher HE144 and HE145 in Israel to *M. zebrinus*. Tree was built using geneious based PHYML plug-in (Dariba et al., 2012). Model of evolution GTR+G+I (Generalised time-reversible model with a proportion of invariable sites and Gamma-distributed among-site rate variation)
