# Supplementary material for: Molecular markers reveal diversity in composition of Megastigmus (Hymenoptera: Megastigmidae) from eucalypt galls
Source: Ecol Evol. 2020 Sep 25;10(20):11565–78. doi: 10.1002/ece3.6791 (PMC7593149; doi:10.1002/ece3.6791)
Supplement: Supplementary file 9 — Appendix S9 [file ECE3-10-11565-s009.docx]

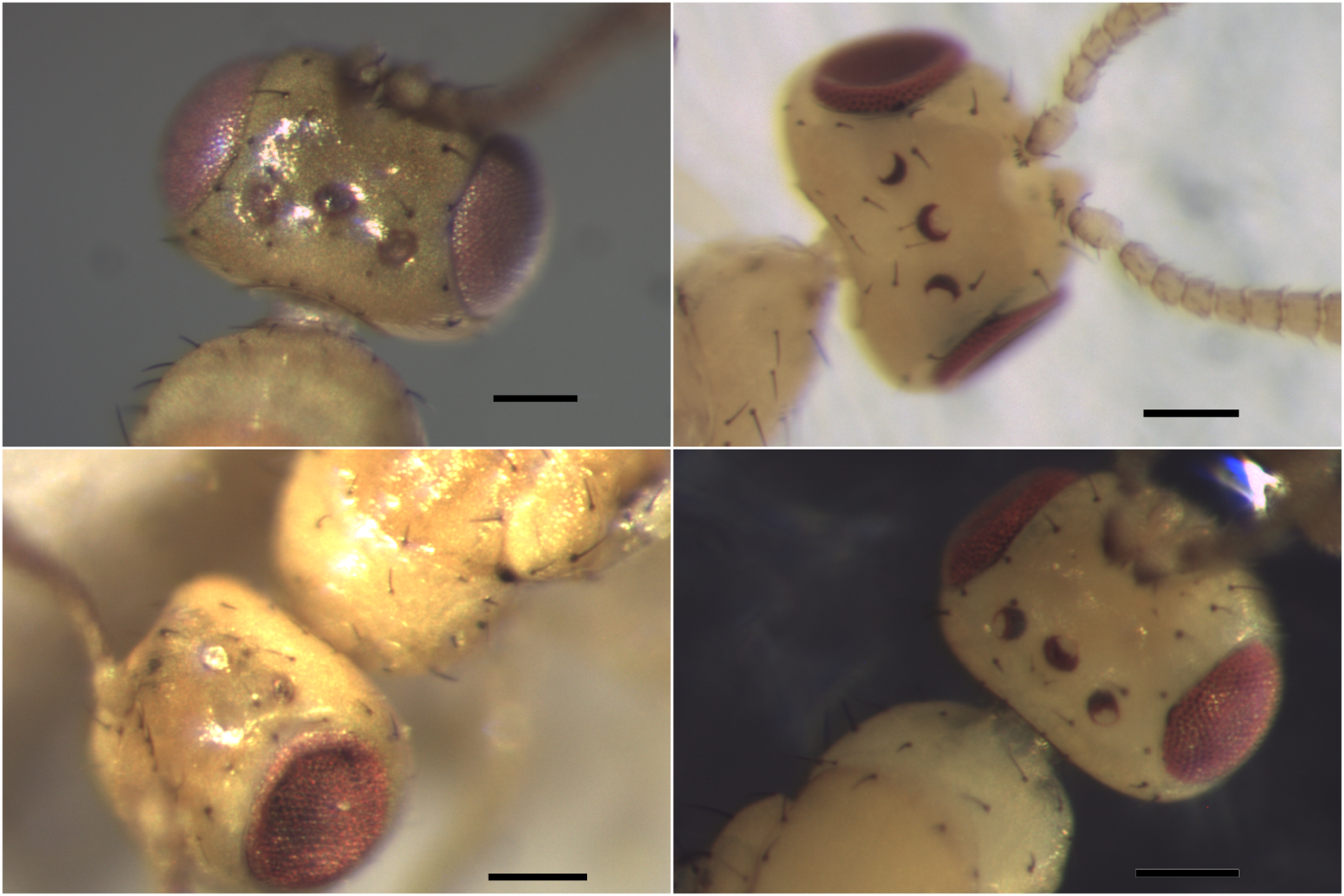


**Supplementary document 9.** Interocellar region of *M. zebrinus*, *M. leptocybus* and *Megastigmus* sp. (Bet She’an valley, Israel). A: *M. zebrinus* paratype ANIC111471; B. Alcohol preserved *Megastigmus* sp., Bet She’an valley, Israel, provided by Dr. Zvi Mendel, 2017. C. *Megastigmus leptocybus* paratype ANIC111467 (one in two females on the same card); D. Alcohol preserved *Megastigmus zebrinus* from South Africa, 2013, provided by Dr. Gudrun Dittrich-Schröder. Scale bar approximate 0.1mm.
